# Five New Polyoxypregnane Glycosides from the Vines of *Aspidopterys* *obcordata* and Their Antinephrolithiasis Activity

**DOI:** 10.3390/molecules27144596

**Published:** 2022-07-19

**Authors:** Zhaocui Sun, Meiying Chen, Qinglong Li, Guoxu Ma, Haifeng Wu, Junshan Yang, Yihang Li, Xudong Xu

**Affiliations:** 1 Key Laboratory of Bioactive Substances and Resource Utilization of Chinese Herbal Medicine, Ministry of Education, Institute of Medicinal Plant Development, Peking Union Medical College, Chinese Academy of Medical Sciences, Beijing 100193, China; flydancingsun@163.com (Z.S.); myc1091@163.com (M.C.); 17861506903@163.com (Q.L.); mgxfl8785@163.com (G.M.); hfwu@implad.ac.cn (H.W.); junshanyang@sina.com (J.Y.); 2 Yunnan Branch, Institute of Medicinal Plant, Chinese Academy of Medical Sciences, Peking Union Medical College, Jinghong 666100, China

**Keywords:** *Aspidopterys obcordata*, polyoxypregnane glycosides, antinephrolithiasis, HK-2 cells

## Abstract

From the dried vines of *Aspidopterys obcordata* Hemsl, five new polyoxypregnane glycosides, named obcordatas J–N (**1**–**5**), were obtained. Their structures were fully elucidated and characterized by HRESIMS and extensive spectroscopic data. In addition, all of the new compounds were screened for their antinephrolithiasis activity in vitro. The results showed that compounds **1**–**3** have prominent protective effects on calcium oxalate crystal-induced human kidney 2 (HK-2) cells, with EC_50_ values ranging from 6.72 to 14.00 μM, which is consistent with the application value of *A. obcordata* in folk medicine for kidney stones.

## 1. Introduction

Pregnane glycosides are substances with a basic steroidal skeleton and at least one glycosidic bond structure [1,2,3]. Such glycosides not only have diverse structures but also show various biological activities, such as anti-tobacco mosaic virus [4], anti-inflammatory [5], antiproliferative [6], antioxidant [7], antibacterial [8], antifungal [9] and antitumor activities [10]. In the last few years, many pregnane glycosides have attracted considerable attention from pharmacologists on account of their remarkable cancer inhibitory or anticarcinogenic activities [11,12,13,14,15]. To date, hundreds of different pregnane glycosides have been found in the plants of Malpighiaceae, Asclepiadaceae, Apocynaceae, Ranunculaceaem and Zygophyllaceae [3,16,17,18,19,20].

**Figure 1:**
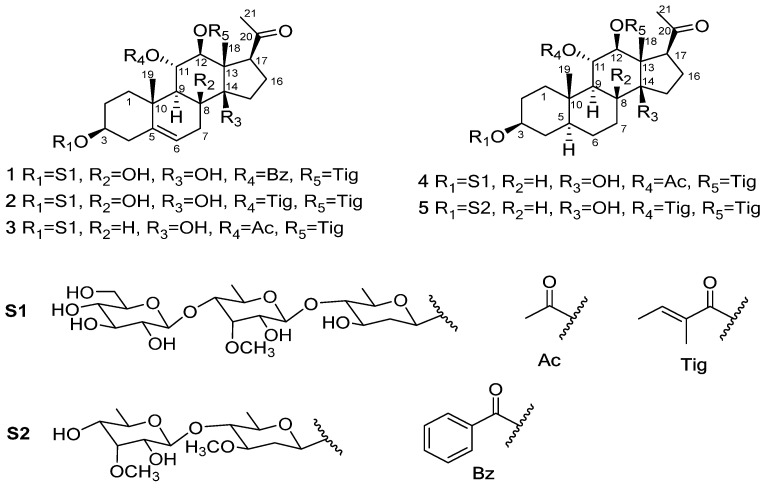
Structures of compounds **1**–**5**.

*A*. *obcordata*, a wood liana of the family Malpighiaceae, is distributed mainly in Xishuangbanna, Yunnan Province, China. The vines of this plant have a long history as a “Dai Medicine” for the treatment of urinary tract infections, chronic nephritis, rheumatic bone pain, cystitis and kidney stones [21,22]. In our previous research, the antinephrolithiasis effects of a distinct polar extract of *A. obcordata* were investigated. The results showed that the 95% ethanol extract of this plant could reduce the volume of kidney stones and decrease urea nitrogen levels and serum creatinine in rats with nephrolithiasis [23]. Although *A*. *obcordata* has been indicated as safe and effective in the treatment of kidney stones, the material basis of this plant’s antinephrolithiasis effect is still unclear. In order to further define the active ingredients, an investigation of the 95% ethanol extracts of the dried vines of *A*. *obcordata* was carried out. Finally, five new polyoxypregnane glycosides, obcordatas J–N (**1**–**5**) (Figure 1), were obtained in the experiment. Thus, this article reports the isolation process and full structural elucidation of these glycosides, as well as their antinephrolithiasis activity in vitro.

## 2. Results

### 2.1. Structure Determination

Compound **1** was obtained as a white amorphous powder, and its molecular formula was inferred to be C_52_H_74_O_20_ from the HRESIMS ion peak at *m*/*z* 1041.4652 [M + Na]^+^ (calcd.1041.4666, C_52_H_74_O_20_Na). Its IR spectrum showed absorption bands ascribed to the hydroxyl (3390 cm^−1^) and carbonyl (1718 cm^−1^) groups. With the assistance of the HSQC spectrum (Supplementary Figure S4), the ^1^H- and ^13^C-APT spectral data of **1** (Table 1 and Table 2) displayed two angular methyl proton signals [*δ*_H_ 1.18 (3H, s, H_3_-18) and 1.14 (3H, s, H_3_-19)], one acetyl methyl proton signal [*δ*_H_ 2.06 (3H, s, H_3_-21)] and one olefic proton signal [*δ*_H_ 5.29 (1H, d, *J* = 5.4 Hz, H-6)], as well as three downfield carbon signals [*δ*_C_ 138.3 (C-5), 118.4 (C-6) and 211.6 (C-20)], which suggests the presence of a C_21_ steroidal pregen-5-en-20-one skeleton in the molecular structure [24]. In addition, its 1D-NMR spectral data (Supplementary Figures S1 and S2) revealed the presence of a benzoyl group [*δ*_H_ 7.80 (2H, dd, *J* = 7.8, 1.2 Hz, Bz-H-3, 7), 7.62 (1H, t, *J* = 7.4 Hz, Bz-H-5) and 7.49 (2H, t, *J* = 7.2 Hz, Bz-H-4, 6); *δ*_C_ 164.8 (Bz-C-1), 129.6 (Bz-C-2), 129.2 (Bz-C-3, 7), 128.6 (Bz-C-4, 6) and 133.4 (Bz-C-5)], a tigloyl group [*δ*_H_ 6.49 (1H, q, *J* = 7.2 Hz, Tig-H-3), 1.53 (3H, d, *J* = 7.2 Hz, Tig-H_3_-4) and 1.35 (3H, s, Tig-H_3_-5); *δ*_C_ 166.8 (Tig-C-1), 127.2 (Tig-C-2), 138.0 (Tig-C-3), 14.1 (Tig-C-4) and 11.3 (Tig-C-5)] and three anomeric methines [*δ*_H_ 4.60 (1H, d, *J* = 9.6 Hz, Oli-H-1, 4.42 (1H, d, *J* = 7.8 Hz, Allo-H-1) and 4.21 (1H, d, *J* = 7.8 Hz, Glc-H-1); *δ*_C_ 96.7 (Oli-C-1), 101.5 (Allo-C-1) and 104.8 (Glc-C-1)].

**Figure 2:**
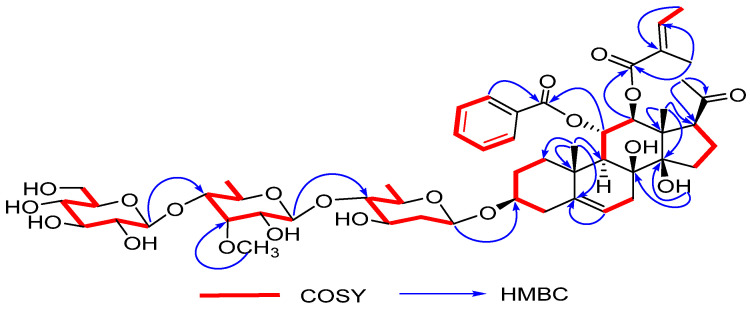
Key ^1^H-^1^H COSY and HMBC correlations for compound **1**.

The ^1^H-^1^H COSY spectrum (Supplementary Figure S3) showed four spin systems, H_2_-1/H_2_-2/H-3/H_2_-4, H-6/H_2_-7, H-9/H-11/H-12 and H_2_-15/H_2_-16/H-17, in the aglycone moiety (highlighted in red in Figure 2). The positions of benzoyl and tigloyl groups were located at C-11 and C-12, respectively, based on HMBC correlations (highlighted in blue in Figure 2) between H-11 (*δ*_H_ 5.88) and Bz-C-1 (*δ*_C_ 164.8), H-12 (*δ*_H_ 5.01) and Tig-C-1 (*δ*_C_ 166.8). Meanwhile, the key HMBC correlations between 8-OH (*δ*_H_ 3.98) and C-8 (*δ*_C_ 75.1) and between 14-OH (*δ*_H_ 4.75) and C-8 (*δ*_C_ 75.1), C-13 (*δ*_C_ 54.6) and C-14(*δ*_C_ 84.5) indicate that two hydroxyl groups are substituted at C-8 and C-14, respectively. Afterwards, according to careful analysis of the 2D NMR spectral data (Supplementary Figures S3–S6), three sugars were proposed to be D-olivopyranose (Oli), 6-deoxy-3-O-methyl-D-allopyranose (Allo) and D-glucose (Glc), further confirmed by TLC and gas chromatography (GC) in comparison with authentic monosaccharides. The connectivity and linkage position of these sugars were identified by their crucial HMBC correlations between Glc-H-1 (*δ*_H_ 4.21) and Allo-C-4 (*δ*_C_ 81.6), between Allo-H-1 (*δ*_H_ 4.42) and Oli-C-4 (*δ*_C_ 87.2), and between Oli-H-1 (*δ*_H_ 4.60) and C-3 (*δ*_C_ 76.8). Based on the above analysis, the planar construction of **1** was determined.

**Figure 3:**
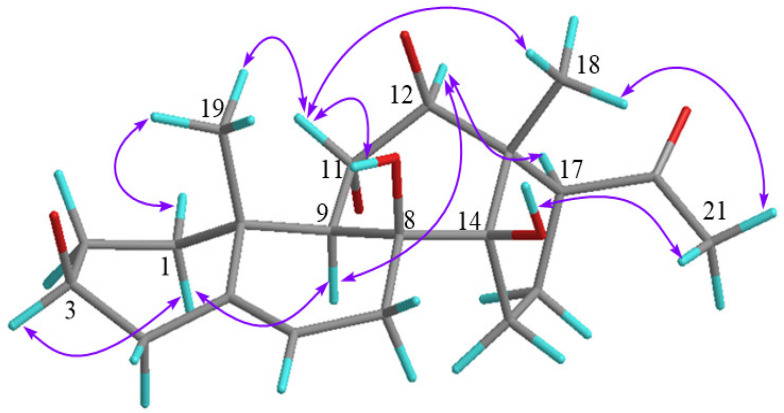
Key nuclear Overhauser effect (NOE) correlations for compound **1**.

The coupling constants and NOESY spectral data (Table 1 and Supplementary Figure S6) were used to clarify the relative configuration of **1**. The β configurations of the three sugars were each confirmed by their large coupling constants (^3^*J*_1,2_ > 7 Hz). Moreover, the coupling constant (*J* = 10.8 Hz) between H-11 and H-12 suggests that both protons are in different directions, which was further verified in the NOESY experiment. Subsequently, the NOE correlations (Figure 3) between H-1*α* and H-3 and H-9, between H-12 and H-17 and H-9, between H_3_-19 and H-11 and H-1*β*, between H_3_-18 and 8-OH, and between H_3_-21 and H_3_-18 and 14-OH indicate that H-3/H-9/H-12/H-17 are *α*-oriented, and H-11/8-OH/14-OH/H_3_-18/H_3_-19 are *β*-oriented. Considering the polyoxypregnane glycosides previously reported for *A. obcordata* [18,24], the absolute configuration of **1** was established. Finally, the whole structure of **1** was identified and named obcordata J.

Compound **2** was suggested to have the molecular formula C_50_H_76_O_20_, as determined by the HRESIMS ion at *m*/*z* 1019.4803 [M + Na]^+^ (calcd. 1019.4822, C_50_H_76_O_20_Na). The ^1^H-NMR and ^13^C-APT spectral data (Table 1 and Table 2, Supplementary Figures S7 and S8) of **2** were structurally similar to those of **1**, except for the absence of the benzoyl group and the presence of an additional tigloyl group in **2**. In the ^13^C-APT spectrum (Supplementary Figure S8) of **2**, the carbon signals at *δ*_C_ 166.9, 165.9, 138.0, 138.0, 128.0, 127.5, 14.3, 14.3, 11.7 and 11.6 suggest the existence of two tigloyl groups in **2**. At the same time, the key HMBC correlations between H-11 (*δ*_H_ 5.69) and 11-O-Tig-C-1 (*δ*_C_ 165.9), H-12 and 12-O-Tig-C-1 (*δ*_C_ 166.9) illustrate that two tigloyl groups are located at C-11 and C-12. Thus, the complete structure of **2** was established and named obcordata K.

Compound **3**, obtained as a white amorphous powder, was established to have the molecular formula C_47_H_72_O_19_ by the HRESIMS ion peak at *m*/*z* 963.4518 [M + Na]^+^ (calcd. 963.4560, C_47_H_72_O_19_Na). Its 1D-NMR data (Supplementary Figures S13 and S14, Table 1 and Table 2) were quite similar to those of **1**, except for the absence of the benzoyl and hydroxy groups and the presence of an extra acetoxyl group [*δ*_H_ 1.80 (3H, s, Ac-H_3_-2); *δ*_C_ 169.6 (Ac-C-1), 21.1 (Ac-C-2)] in **3**. The HMBC correlations between H-11 (*δ*_H_ 5.25) and Ac-C-1 (*δ*_C_ 169.6) indicate that the acetoxyl group is replaced at C-11. Based on the HMBC correlations between 14-OH (*δ*_H_ 4.48) and C-8 (*δ*_C_ 36.8), C-13 (*δ*_C_ 54.0), C-14 (*δ*_C_ 82.9) and C-15 (*δ*_C_ 33.3), the remaining hydroxyl group was identified to be substituted at C-14. Together with its NOESY spectral data (Supplementary Figure S18), the structure of **3** was finally confirmed and named obcordata L. 

Compound **4** was isolated and purified as a white amorphous powder. The HRESIMS displayed an ion peak at *m*/*z* 965.4703 [M + Na]^+^ (calcd. 965.4717, C_47_H_74_O_19_Na), which showed the molecular formula C_47_H_74_O_19_ and two more mass units than obcordata L (**3**). By detailed comparison of the 1D-NMR spectral data (Table 1 and Table 2, Supplementary Figures S19 and S20) with those of **3**, significant differences were the disappearance of one double bond and the presence of an extra methine *δ*_C_ 43.6 (C-5) and one additional methylene *δ*_C_ 29.6 (C-6), which indicates that **4** is the reduction product of **3**. In the NOESY spectrum, correlations between H-3 and H-5, H-5 and H-9 suggest that H-5 is α-oriented. Finally, compound **4** was illustrated and given the name obcordata M.

Compound **5** was suggested to be C_45_H_70_O_14_ based on the HRESIMS pseudomolecular ion peak at *m*/*z* 857.4640 [M + Na]^+^ (calcd. 857.4658, C_45_H_70_O_14_Na). The overall consideration of 1D- (Table 1 and Table 2) and 2D-NMR spectral data (Supplementary Figures S25–S30) suggests the presence of a (5*α*,8*β*,9*α*,17*α*)-20-one-3*β*,11*α*,12*β*,14*β*-tetradroxypregnane aglycone moiety in **5**. With the aid of the HSQC spectrum (Supplementary Figure S28), the ^13^C-APT spectrum (Table 2 and Supplementary Figure S26) of **5** revealed the appearance of two tigloyl groups (*δ*_C_ 166.8, 166.4, 138.0, 138.0, 127.9, 127.5, 14.2, 14.2, 11.7 and 11.6) and two sugar units (two anomeric carbons at *δ*_C_ 100.8 (Allo-C-1) and 96.4 (Ole-C-1)). Two tigloyl groups were confirmed to be located at C-11 and C-12 by the HMBC correlations between H-11 (*δ*_H_ 5.21) and 11-O-Tig-C-1 (*δ*_C_ 166.4) and between H-12 (*δ*_H_ 4.75) and 12-O-Tig-C-1 (*δ*_C_ 166.8). With the assistance of 2D-NMR spectral data, two sugar units were fully assigned as D-oleandrose (Ole) and 6-deoxy-3-O-methyl-D-allopyranose (Allo) after hydrolysis, which are consistent with the sugars of obcordata A previously reported from *A. obcordata* [23]. Based on the NOESY spectral data, compound **5** was finally identified and named obcordata N.

### 2.2. Antinephrolithiasis Activity

**Figure 4:**
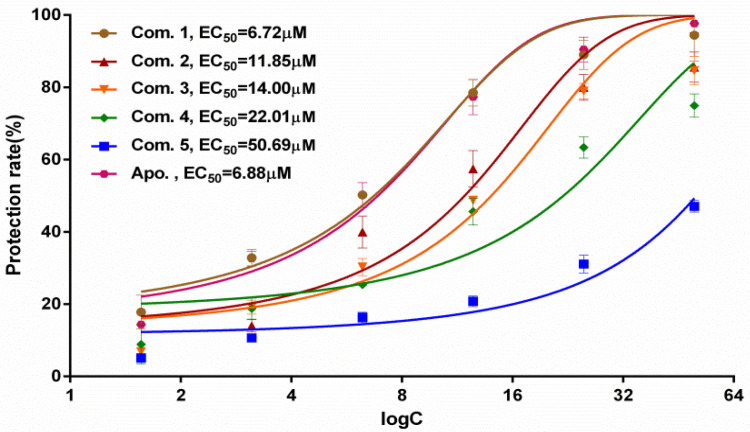
Protective effects of compounds **1**–**5** in HK-2 cells injured by calcium oxalate crystals.

The antinephrolithiasis activity of the obtained compounds **1**–**5** was evaluated in HK-2 cells injured by calcium oxalate crystals via the MTT assay [23,25,26]. In view of the potential cytotoxicity of compounds **1**–**5** in mammalian cells, normal HK-2 cells were treated with 100 μM of all compounds for 24 h, and the cell viabilities were not significantly affected. Afterwards, the protective effects of compounds (**1**–**5**) against calcium oxalate crystal-induced HK-2 cells were determined in vitro. The results of the viabilities of injured HK-2 cells (Figure 4) showed that the EC_50_ of all isolated compounds ranged from 6.72 to 50.69 μM. Among them, compounds **1**–**3** displayed better protection in injured HK-2 cells, with EC_50_ values of 6.72, 11.85 and 14.00 μM, respectively. It is worth noting that compound **1** exhibited the most potent antinephrolithiasis activity, with an EC_50_ value of 6.72 μM, compared with the positive control apocynin (Apo.), with an EC_50_ value of 6.88 μM. Therefore, the protective mechanism of obcordata J (**1**) against nephrolithiasis activity deserves significant further exploration. 

## 3. Materials and Methods

### 3.1. General Experimental Materials

UV spectra and optical rotations were measured with a UV2550 (Shimadzu, Kyoto, Japan) and a 341 digital polarimeter (PerkinElmer, Norwalk, CT, USA), respectively. IR spectral data were determined with FTIR-8400 spectrometers (Shimadzu, Japan). NMR spectral data were recorded on a Bruker AV III 600 NMR spectrometer (Bruker, Billerica, Germany). Mass spectra were performed by using the Waters micromass Q-TOF system (Waters, Bremen, GA, USA). Silica gels (200–300 mesh, Qingdao Marine Chemical Plant, Qingdao, China) were used for column chromatography (CC). TLC analyses were measured by spraying with 5% H_2_SO_4_ and heating at 100 °C (silica gel GF 254, Qingdao Haiyang Chemical Co., Qingdao, China). All solvents (Beijing Chemical Works, Beijing, China) used were analytical grade.

### 3.2. Plant Material

The vines of *A. obcordata* were collected from Jinghong (Yunnan Province, China) and were authenticated by Professor Rongtao Li (Yunnan Branch, Institute of Medicinal Plant (IMPLAD)). The voucher specimen (CS-16368) was deposited at IMPLAD.

### 3.3. Extraction and Isolation

The vines of *A. obcordata* (5.0 kg) were air-dried, powdered and then repeatedly extracted with 95% ethanol (25 L) four times, and each extraction lasted for 3 h. The extracted solution was evaporated under vacuum to provide the crude ethyl alcohol extract (280.0 g). The concentrated 95% ethanol extract was dissolved in water and partitioned successively using different solvents (petroleum ether, dichloromethane, ethyl acetate and n-butanol, 2 L, 3 times) to obtain different extracts. The ethyl acetate fraction (80 g) was selected for further separation on account of its moderate antinephrolithiasis effect on HK-2 cells exposed to calcium oxalate crystals [27]. The crude ethyl acetate extracts were chromatographed over silica gel CC (200–300 mesh, 10 cm × 100 cm) using dichloromethane–methanol (1:0→0:1, 5 L), which yielded 12 fractions. Semi-preparative HPLC (S-HPLC) with a Lumtech K-1001 analytic HPLC system (a K-2600 UV detector and two K-501 pumps) and a YMC Pack C_18_ column (250 mm × 10 mm, 5 μM, YMC Co. Ltd., Kyoto, Japan) was used to purify compounds (methanol–water system, 2 mL/min). Fr.8 (5.6 g) was purified by S-HPLC (methanol–water system, 72:28, *v*/*v*) to yield obcordata M (**4**)(1.5 mg, t_R_ = 18.5 min) and obcordata N (**5**) (1.6 mg, t_R_ = 25.2 min). Fr.9 (6 g) was purified by S-HPLC (methanol–water system, 52:48, *v*/*v*) to yield obcordata J (**1**) (4 mg, t_R_ = 13.4 min), obcordata K (**2**) (1.9 mg, t_R_ = 16.2 min) and obcordata L (**3**) (1.8 mg, t_R_ = 19.3 min).

### 3.4. Characterization of Compounds **1**–**5**

Obcordata J (**1**): White amorphous powder; [*α*]^25^_D_ +37 (c 0.15, methanol); UV λ_max_ (methanol) (log *ε*): 275 (3.62) nm; IR (KBr) *ν*_max_: 3,423, 1718, 1660, 1512, 1420 and 1210 cm^−1^; HRESIMS *m*/*z* 1041.4652 [M + Na]^+^ (calculated for C_52_H_74_O_20_Na, 1041.4666,); ^1^H- and ^13^C-NMR spectral (600, 150 MHz, DMSO-*d_6_*) data: see Table 1 and Table 2.

Obcordata K (**2**): White amorphous powder; [*α*]^25^_D_ +40.5 (c 0.10, methanol); UV λ_max_ (methanol) (log *ε*): 275 (3.35) nm; IR (KBr) *ν*_max_: 3390, 1715, 1658, 1503, 1427 and 1215 cm^−1^; HRESIMS *m*/*z* 1019.4803 [M + Na]^+^ (calculated for C_50_H_76_O_20_Na, 1019.4822); ^1^H- and ^13^C-NMR spectral (600, 150 MHz, DMSO-*d_6_*) data: see Table 1 and Table 2.

Obcordata L (**3**): White amorphous powder; [*α*]^25^_D_ +48.2 (c 0.11, methanol); UV λ_max_ (methanol) (log *ε*): 272 (3.04) nm; IR (KBr) *ν*_max_: 3403 and 1720 cm^−1^; HRESIMS *m*/*z* 963.4518 [M + Na]^+^ (calculated for C_47_H_72_O_19_Na, 963.4560); ^1^H- and ^13^C-NMR spectral (600, 150 MHz, DMSO-*d_6_*) data: see Table 1 and Table 2.

Obcordata M (**4**): White amorphous powder; [*α*]^25^_D_ +52.2 (c 0.13, methanol); UV λ_max_ (MeOH) (log *ε*): 225 (2.84) nm; IR (KBr) *ν*_max_: 3410, 1718; 1636 and 1524 cm^−1^; HRESIMS *m*/*z* 965.4703 [M + Na]^+^ (calculated for C_47_H_74_O_19_Na, 965.4717); ^1^H- and ^13^C-NMR spectral (600, 150 MHz, DMSO-*d_6_*) data: see Table 1 and Table 2.

Obcordata N (**5**): White amorphous powder; [*α*]^25^_D_ +44.6 (c 0.16, methanol); UV λ_max_ (methanol) (log *ε*): 230 (2.90) nm; IR (KBr) *ν*_max_: 3385, 1690, 1620 and 1542 cm^−1^; HRESIMS *m*/*z* 857.4640 [M + Na]^+^ (calculated for C_45_H_70_O_14_Na, 857.4658); ^1^H- and ^13^C-NMR spectral (600, 150 MHz, DMSO-*d_6_*) data: see Table 1 and Table 2.

### 3.5. Compound Hydrolysis 

The hydrolysis method of compounds **1**–**5** was the same as reported in our previous study [24].

### 3.6. Cytotoxicity Assay 

In 96-well microplates, HK-2 cells were seeded (2 × 10^4^ cells/mL) and treated with compounds **1**–**5** in different concentrations (6.25, 12.5, 25, 50 and 100 µM) for 24 h. After that, the cytotoxicity of all compounds against HK-2 cells was determined via the MTT assay. Furthermore, an automatic multifunctional microplate reader was used to measure the OD values at 570 nm.

### 3.7. Antinephrolithiasis Screening 

The method of preparing COM (calcium oxalate monohydrate) crystals was used with little modification, as described previously [23,25,26]. Different concentrations (1.56, 3.12, 6.25, 12.5, 25 and 50 µM) of compounds **1**–**5** were used in the dose-dependent study using this method. Cells without any treatment were used as a control. Furthermore, various concentrations of Apo. were used as a positive control. After 24 h, the viability of the injured HK-2 cells was measured by the MTT assay. 

## 4. Conclusions

In this study, five new polyoxypregnane glycosides, obcordatas J–N (**1**–**5**), were obtained from 95% EtOH extracts of the dried vines of *A. obcordata*. The complete structures of all of these new compounds were eventually elucidated by extensive spectral analysis. Their antinephrolithiasis activities were measured based on the viability of HK-2 cells exposed to COM crystals in vitro. Among all of the tested compounds, obcordata J (**1**) was revealed to have the most potent antinephrolithiasis activity, with an EC_50_ value of 6.72 μM. To date, about 60 compounds, including 17 polyoxypregnane glycosides, have been isolated from *A. obcordata*. Interestingly, the glycoside obcordata A was reported to exert its antinephrolithiasis activity through the NOX4/ROS/P38 MAPK pathway [18,22,23,24,28]. In sum, polyoxypregnane glycosides might be the material basis of the *A. obcordata* antinephrolithiasis effect, which deserves in-depth study.

## Figures and Tables

**Table 1:** ^1^H-NMR (600 MHz) spectral data of compounds **1**–**5** in DMSO-*d_6_*.

Position	1	2	3	4	5
1	1.17 (m), 1.81 (m)	1.17 (m), 1.81 (m)	1.17 (m), 1.81 (m)	1.20 (m), 1.71 (m)	0.92 (m), 1.90 (m)
2	1.71 (m), 1.99 (m)	1.71 (m), 2.00 (m)	1.61 (m), 2.01 (m)	1.75 (m), 1.99 (m)	1.59 (m), 2.03 (m)
3	3.01 (m)	3.06 (m)	3.06 (m)	3.01 (m)	3.26 (m)
4	2.21 (m), 2.43 (m)	2.21 (m), 2.42 (m)	2.21 (m), 2.31 (m)	1.59 (m), 1.62 (m)	1.44 (m), 2.09 (m)
5	-	-	-	1.18 (m)	1.90 (m)
6	5.29 (d, *J* = 5.4 Hz)	5.17 (d, *J* = 5.4 Hz)	5.45 (d, *J* = 5.4 Hz)	1.07 (m), 1.61 (m)	1.04 (m), 1.66 (m)
7	1.81 (m), 2.24 (m)	1.88 (m), 2.21 (m)	1.17 (m), 2.11 (m)	1.42 (m), 1.60 (m)	1.36 (m), 1.37 (m)
8	-	-	1.80 (m)	2.06 (m)	2.06 (m)
9	2.01 (d, *J* = 10.8 Hz)	1.96 (d, *J* = 10.2 Hz)	1.63(d, *J* = 10.2 Hz)	1.65 (d, *J* = 10.8 Hz)	1.66 (d, *J* = 10.2 Hz)
10	-	-	-	-	-
11	5.89 (t, *J* = 10.8 Hz)	5.70 (t, *J* = 10.2 Hz)	5.25 (t, *J* = 10.2 Hz)	5.16 (t, *J* = 10.2 Hz)	5.21 (t, *J* = 10.8 Hz)
12	5.02 (d, *J* = 10.8 Hz)	4.89 (d, *J* = 10.2 Hz)	4.78 (d, *J* = 10.2 Hz)	4.97 (d, *J* = 10.2 Hz)	4.75 (d, *J* = 10.8 Hz)
13	-	-	-	-	-
14	-	-	-	-	-
15	1.81 (m), 2.21 (m)	1.85 (m), 2.21 (m)	1.21 (m), 1.31 (m)	1.41 (m), 1.53 (m)	1.49 (m), 1.56 (m)
16	1.80 (m), 2.11 (m)	1.79 (m), 2.11 (m)	1.39 (m), 2.41 (m)	1.81 (m), 2.19 (m)	1.81 (m), 2.12 (m)
17	2.70 (m)	2.89 (m)	3.34 (m)	3.35 (m)	3.34 (m)
18	1.14 (s)	1.22 (s)	0.96 (s)	0.81(s)	0.82 (s)
19	1.35 (s)	1.43 (s)	1.01 (s)	0.91 (s)	0.92 (s)
20	-	-	-	-	-
21	2.13 (s)	2.15 (s)	2.06 (s)	2.10 (s)	2.07 (s)
11-O	Bz	Tig	Ac	Ac	Tig
2	-	-	1.80 (s)	1.78 (s)	-
3	7.80 (dd, *J* = 7.2, 1.2 Hz)	6.61 (q, *J* = 7.2 Hz)	-	-	6.58 (q, *J* = 7.2 Hz)
4	7.49 (t, *J* = 7.2 Hz)	1.73 (d, *J* = 7.2 Hz)	-	-	1.69 (d, *J* = 7.2 Hz)
5	7.62 (t, *J* = 7.2 Hz)	1.61 (s)	-	-	1.60 (s)
6	7.49 (t, *J* = 7.2 Hz)	-	-	-	-
7	7.80 (dd, *J* = 7.2, 1.2 Hz)	-	-	-	-
12-O	Tig	Tig	Tig	Tig	Tig
2	-	-	-	-	-
3	6.49 (q, *J* = 7.2 Hz)	6.69 (q, *J* = 7.2 Hz)	6.83 (q, *J* = 7.2 Hz)	6.81 (q, *J* = 7.2 Hz)	6.68 (q, *J* = 7.2 Hz)
4	1.53 (d, J = 7.2 Hz)	1.74 (d, J = 7.2 Hz)	1.80 (d, J = 7.2 Hz)	1.79 (d, J = 7.2 Hz)	1.73 (d, *J* = 7.2 Hz)
5	1.35 (s)	1.69 (s)	1.78 (s)	1.76 (s)	1.60 (s)
Oli/Ole-1	4.60 (d, *J* = 9.6 Hz)	4.62 (d, *J* = 9.6 Hz)	4.78 (d, *J* = 9.6 Hz)	4.65 (t, *J* = 9.6 Hz)	4.56 (t, *J* = 9.6 Hz)
2	1.82 (m), 2.12 (m)	1.82 (m), 2.23 (m)	1.82 (m), 2.21 (m)	1.42 (m), 2.21 (m)	1.91 (m), 2.25 (m)
3	3.01 (m)	3.42 (m)	3.52 (m)	3.02 (m)	3.47 (m)
4	3.85 (m)	3.86 (m)	3.21 (m)	3.82 (m)	3.37 (m)
5	3.14 (m)	3.01 (m)	3.01 (m)	3.00 (m)	3.45 (m)
6	1.22 (d, *J* = 6.0 Hz)	1.19 (d, *J* = 6.0 Hz)	1.24 (d, *J* = 6.0 Hz)	1.19 (d, *J* = 6.0 Hz)	1.09 (d, *J* = 6.0 Hz)
-OCH_3_	-	-	-	-	3.26 (s)
Allo-1	4.42 (d, *J* = 7.8 Hz)	4.45 (d, *J* = 8.4 Hz)	4.48 (d, *J* = 7.8 Hz)	4.46 (d, *J* = 7.8 Hz)	4.55 (d, *J* = 7.8 Hz)
2	3.21 (m)	3.22 (m)	3.01 (m)	3.24 (m)	3.15 (m)
3	3.82 (m)	3.81 (m)	3.81 (m)	3.85 (m)	3.04 (m)
4	3.38 (m)	3.61 (m)	3.33 (m)	3.25 (m)	3.34 (m)
5	3.61 (m)	3.61 (m)	3.61 (m)	3.27 (m)	3.26 (m)
6	1.21 (d, *J* = 6.0 Hz)	1.23 (d, *J* = 6.0 Hz)	1.20 (d, *J* = 6.0 Hz)	1.23 (d, *J* = 6.0 Hz)	1.23 (d, *J* = 6.6 Hz)
3-OCH_3_	3.74 (s)	3.74 (s)	3.46 (s)	3.46 (s)	3.47 (s)
Glc-1	4.21(d, *J* = 7.8 Hz)	4.22(d, *J* = 7.8 Hz)	4.22(d, *J* = 7.8 Hz)	4.22(d, *J* = 7.8 Hz)	-
2	2.89 (m)	2.89 (m)	2.99 (m)	3.24 (m)	-
3	3.05 (m)	3.02 (m)	3.15 (m)	3.05 (m)	-
4	3.40 (m)	3.41 (m)	3.01 (m)	3.04 (m)	-
5	3.31 (m)	3.24 (m)	3.71 (m)	3.19 (m)	-
6	3.51 (m)	3.74 (m)	3.50 (m)	3.46 (m)	-
8-OH/14-OH	3.98 (s)/4.75 (s)	3.90 (s)/4.71 (s)	-/4.48 (s)	-/4.42 (s)	-/4.40 (s)

**Table 2:** ^13^C-NMR (150 MHz) spectral data of compounds **1**–**5** in DMSO-*d_6_*.

Position	1	2	3	4	5	Position	1	2	3	4	5
1	38.0	38.0	37.8	37.0	37.0	7	129.2	-	-	-	-
2	29.0	29.1	29.4	27.5	27.5	12-O	Tig	Tig	Tig	Tig	Tig
3	76.8	76.6	76.6	76.6	75.2	1	166.8	166.9	166.9	166.8	166.8
4	38.6	38.1	38.4	33.6	32.7	2	127.2	127.5	127.4	127.5	127.5
5	138.4	138.4	139.0	43.6	43.6	3	138.0	138.0	138.7	138.5	138.0
6	118.4	118.3	121.9	29.6	29.4	4	14.1	14.3	14.4	14.4	14.2
7	26.0	26.2	27.1	28.6	28.6	5	11.3	11.6	11.9	11.8	11.7
8	75.1	75.0	36.8	36.5	36.6	Oli/Ole-1	96.7	96.8	96.8	96.5	96.4
9	47.6	47.6	46.5	48.7	48.9	2	38.9	38.9	38.9	38.7	36.6
10	39.2	39.3	39.0	37.1	37.1	3	68.8	68.8	68.8	68.8	78.6
11	71.4	70.5	70.5	70.8	70.8	4	87.2	87.2	87.2	87.3	82.3
12	77.6	77.6	77.0	77.4	77.5	5	69.9	69.9	70.0	69.9	70.5
13	54.6	54.6	54.0	54.0	53.9	6	17.2	17.2	17.2	17.2	18.0
14	84.5	84.5	82.9	82.8	82.9	-OCH_3_	-	-	-	-	56.3
15	35.5	35.5	33.3	34.6	34.6	Allo-1	101.5	101.6	101.6	101.6	100.8
16	23.2	22.8	22.8	23.0	23.1	2	70.5	70.5	70.5	70.5	73.1
17	58.6	58.6	57.8	57.8	57.8	3	81.4	81.4	81.4	81.4	82.8
18	12.9	12.9	11.4	11.5	11.5	4	81.6	81.6	81.6	81.6	71.6
19	17.6	17.2	18.6	13.6	11.7	5	68.6	68.6	68.6	68.6	69.4
20	211.6	211.5	211.1	211.1	211.1	6	17.3	17.7	17.7	17.7	18.4
21	30.6	30.6	30.7	30.7	30.7	3-OCH_3_	60.9	61.0	61.0	61.0	61.4
11-O	Bz	Tig	Ac	Ac	Tig	Glc-1	104.8	104.9	104.9	104.9	-
1	164.8	165.9	169.6	169.8	166.4	2	76.6	76.6	76.6	76.6	-
2	129.6	128.0	21.1	21.2	127.9	3	73.7	73.7	73.7	73.7	-
3	129.2	138.0	-	-	138.0	4	70.1	70.2	70.2	70.2	-
4	128.6	14.3	-	-	14.2	5	76.9	76.9	77.0	76.9	-
5	133.4	11.7	-	-	11.6	6	61.4	61.4	61.4	61.4	-
6	128.6	-	-	-	-						

## Data Availability

The data presented in this study are available in the Supplementary Materials.

## Supplementary Materials

The following supporting information can be downloaded at: https://www.mdpi.com/article/10.3390/molecules27144596/s1. Figures S1–S30: ^1^H-NMR, ^13^C-APT, ^1^H-^1^H COSY, HSQC, HMBC and NOESY spectral data for compounds (**1**–**5**).

## Sample Availability

Samples of the compounds are not available from the authors.

## References

[1] Reichstein T. (1967). Cardenolid-und pregnanglykoside. Naturwissenwdhaftern.

[2] Gupta M., Gupta V., Khare N.N. (2015). Structural studies of biologically importantsteroidal pregnane glycosides from medicinal plants. Trends Carbohydr. Res..

[3] Si Y., Sha X.S., Shi L.L., Wei H.Y., Jin Y.X., Ma G.X., Zhang J. (2022). Review on pregnaneglycosides and their biological activities. Phytochem. Lett..

[4] Yan Y., Zhang J.X., Liu K.X., Huang T., Yan C., Huang L.J., Liu S., Mu S.Z., Hao X.J. (2014). Seco-pregnane steroidal glycosides from the roots of *Cynanchumatratum* andtheir anti-TMV activity. Fitoterapia.

[5] Lee C.L., Hwang T.L., He W.J., Tsai Y.H., Yen C.T., Yen H.F., Chen C.J., Chang W.Y., Wu Y.C. (2013). Anti-neutrophilic inflammatory steroidal glycosides from *Solanumtorvum*. Phytochemistry.

[6] Plaza A., Perrone A., Balestrieri M.L., Felice F., Balestrieri C., Hamed A.I., Pizza C., Piacente S. (2005). New unusual pregnane glycosides with antiproliferative activity from *Solenostemma*
*argel*. Steroids.

[7] Ounaissia K., Pertuit D., Mitaine-offer A.C., Miyamoto T., Tanaka C., Delemasure S.P., Dutartre P., Smati D., Lacaille-dubois M.A. (2016). New pregnane and phenolic glycosides from *Solenostemmaargel*. Fitoterapia.

[8] Song C.W., Lung P.K., Qin X.J., Cheng G.G., Gu J.L., Liu Y.P., Luo X.D. (2014). New antimicrobial pregnane glycosides from the stem of *Ecdysantherarosea*. Fitoterapia.

[9] Zhao D., Feng B., Chen S., Chen G., Li Z., Li X., Sang X., An X., Wang H., Pei Y. (2016). C_21_ steroidal glycosides from the roots of *Cynanchum*
*paniculatum*. Fitoterapia.

[10] Huang L.J., Wang B., Zhang J.X., Yan C., Mu S.Z., Hao X.J. (2015). Studies oncytotoxic pregnane sapogenins from *Cynanchumwilfordii*. Fitoterapia.

[11] Liu X., Zhang Y., Huang W., Luo J., Li Y., Tan W., Zhang A. (2018). Development of high potent and selective Bcl-2 inhibitors bearing the structural elements of natural product artemisinin. Eur. J. Med. Chem..

[12] Song J., Dai R., Deng Y., Lv F. (2018). Rapid structure prediction by HPLC-ESI-MS of twenty-five polyoxypregnane tetraglycosides from *Dregea*
*Sinensis* with NMR confirmation of eight structures. Phytochemistry.

[13] Zhang X., Zhou Y., Zuo J., Yu B. (2015). Total synthesis of periploside A, aunique pregnane hexasaccharide with potent immunosuppressive effects. Nat. Commun..

[14] Yao S., To K.K.-W., Wang Y.-Z., Yin C., Chai S., Ke C.Q., Lin G., Ye Y. (2014). Polyoxypregnane steroids from the stems of *Marsdenia*
*tenacissima*. J. Nat. Prod..

[15] Sun D.F., Sun J.Y., Fan H.X., Yao Q.Q. (2014). Advances in studies on C_21_ steroidal glycosides of plants in *Asclepiadaceae*. Chin. Trad. Herbal Drugs.

[16] Liu S.Z., Chen Z.H., Wu J., Wang L.Y., Wang H.M., Zhao W.M. (2013). Appetite suppressing pregnane glycosides from the roots of *Cynanchum*
*auriculatum*. Phytochemistry.

[17] Tatsuno S., Yokosuka A., Hatauma F., Mashiko Y., Mimaki Y. (2019). Pregnaneglycosides from the bark of *Marsdenia*
*cundurango* and their cytotoxic activity. J. Nat. Med..

[18] Guo H.W., Tian Y.G., Liu Y.H., Huang J., Wang J.X., Long H., Wei H. (2021). Discovery of polyoxypregnane derivatives from *Aspidopterys*
*obcordata* with their potential antitumor activity. Front. Chem..

[19] Minpei K., Satoshi K., Daichi M., Yukiko M., Hiroshi S., Yoshihiro M. (2018). Aestivalosides A–L, twelve pregnane glycosides from the seeds of *Adonis aestivalis*. Phytochemistry.

[20] Zhao D., Su S.S., Chen S.F., Lu X., Chen G., Wang Y.B., Su G.Y., Pei Y.H. (2018). Two new C_21_ steroidal glycosides isolated from *Cynanchum*
*komarovii*. Chin. J. Nat. Med..

[21] Wu R., Ye Q., Chen N., Zhang G. (2001). Study on the chemical constituents of *Aspidopterys*
*obcordata* Hemsl. Nat. Prod. Rsc. Dev..

[22] Li H., Peng C.Z., Guan Y.H., Niu Y.F., Zhang L.X. (2011). Resources investigation on *Aspidopterys*
*obcordata*. Shi Zhen Guo Yi Guo Yao.

[23] Li Y., Ma G., Lv Y., Su J., Li G., Chen X. (2019). Efficacy of Obcordata A from *Aspidopterys obcordata* on Kidney Stones by Inhibiting NOX_4_ Expression. Molecules.

[24] Hu M.G., Li Y.H., Sun Z.C., Huo X.W., Zhu N.L., Sun Z.H., Liu Y.Y., Wu H.F., Xu X.D., Ma G.X. (2018). New polyoxypregnane glycosides from *Aspidopterys*
*obcordata* vines with antitumor activity. Fitoterapia.

[25] Li S., Liu Q.-Q. (2021). A study on the mechanism of the protective effect of GuangeFang on sepsis-associated acute kidney injury. World J. Tradit. Chin. Med..

[26] Wiessner J.H., Hung L.Y., Mandel N.S. (2003). Crystal attachment to injured renal collecting duct cells: Influence of urine proteins and pH. Kidney Int..

[27] Song M.F., Li Y.H., Zhang Z.L., Lv Y.N., Li X.L., Li G. (2015). Inhibiting effect of *Aspidopterys obcordata* Hemsl on renal calculus. Chin. J. New Drugs.

[28] Han Y., Hao M.M., Ruan J.Y., Bai Y., Wang T., Zhang Y. (2020). Research Progress on Chemical Constituents and Pharmacological Effects of *Aspidopterys Obcordata* Hemsl. Chin. J. Ethomed. Ethnopharm..

