# Supplementary material for: Five New Polyoxypregnane Glycosides from the Vines of Aspidopterys obcordata and Their Antinephrolithiasis Activity
Source: Molecules. 2022 Jul 19;27(14):4596. doi: 10.3390/molecules27144596 (PMC9324186; doi:10.3390/molecules27144596)
Supplement: Supplementary file 1 [file molecules-27-04596-s001.zip › molecules-1800014-supplementary.pdf]

Supporting information for

Five New Polyoxypregnane Glycosides from the Vines of *Aspidopterys obcordata* and Their Antinephrolithiasis Activity

Zhaocui Sun [1,†], Meiying Chen [1,†], Qinglong Li [1], Guoxu Ma [1], Haifeng Wu [1], Junshan Yang [1], Yihang Li [1,2,*] and Xudong Xu [1,*]

1 Key Laboratory of Bioactive Substances and Resource Utilization of Chinese Herbal Medicine, Ministry of Education, Institute of Medicinal Plant Development, Peking Union Medical College, Chinese Academy of Medical Sciences, Beijing 100193, China; flydancingsun@163.com (Z.S.); myc1091@163.com (M.C.); 17861506903@163.com (Q.L.); mgxfl8785@163.com (G.M.); hfwu@implad.ac.cn (H.W.); junshanyang@sina.com (J.Y.)

2 Yunnan Branch, Institute of Medicinal Plant, Chinese Academy of Medical Sciences, Peking Union Medical College, Jinghong 666100, China

* Correspondence: yhli@implad.ac.cn (Y.L.); xdxu@implad.ac.cn (X.X.)

† These authors contributed equally to this work.

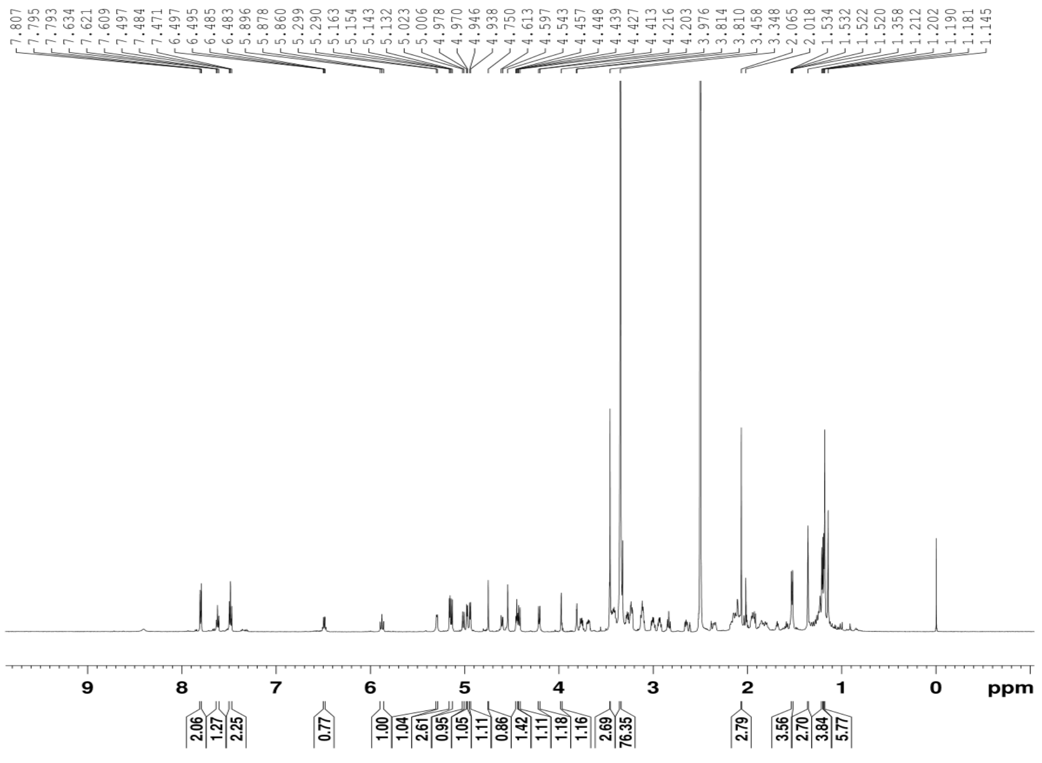

Figure S1. ^{1}H-NMR spectrum of obcordata J (**1**) (600 MHz, DMSO-d_6)

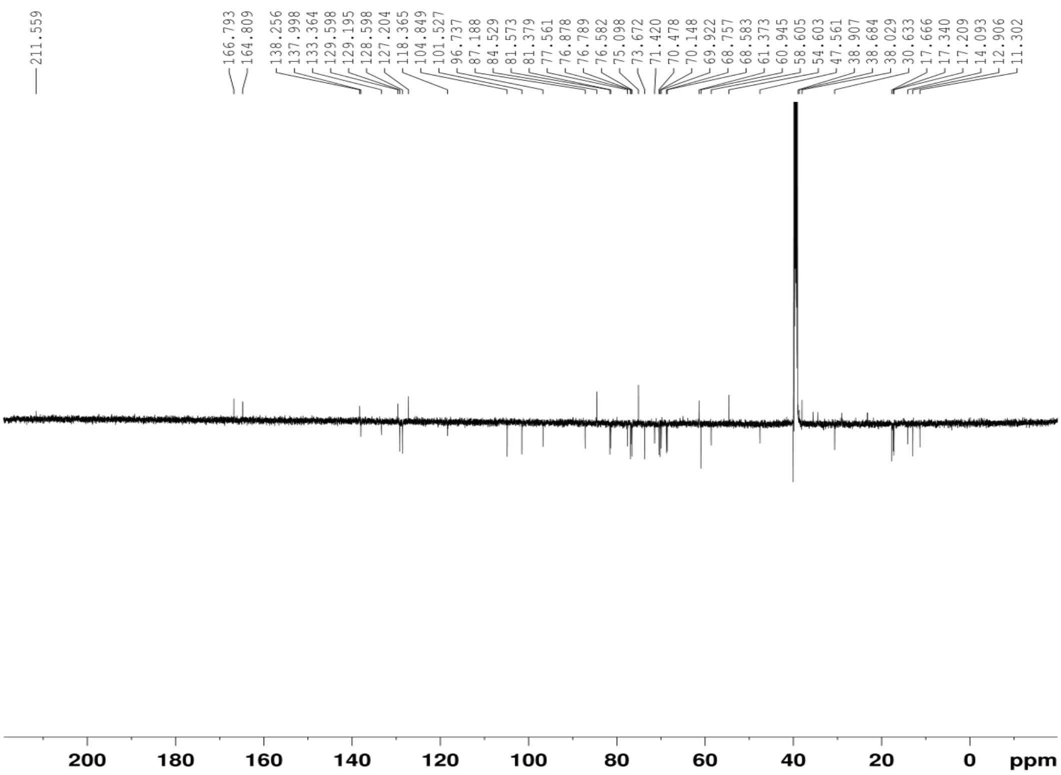

Figure S2. ^{13}C-APT spectrum of obcordata J (**1**) (150 MHz, DMSO-d_6)

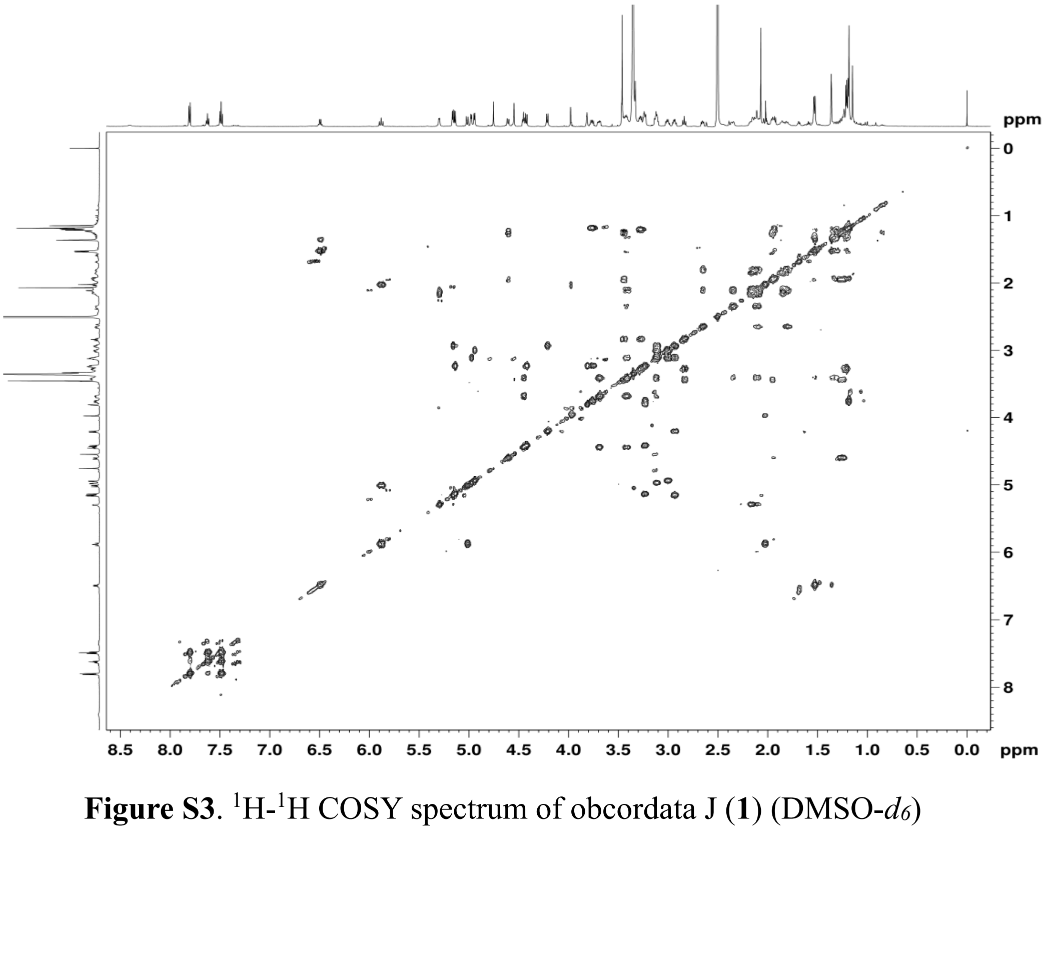

Figure S3. ^{1}H-^{1}H COSY spectrum of obcordata J (**1**) (DMSO-d_6)

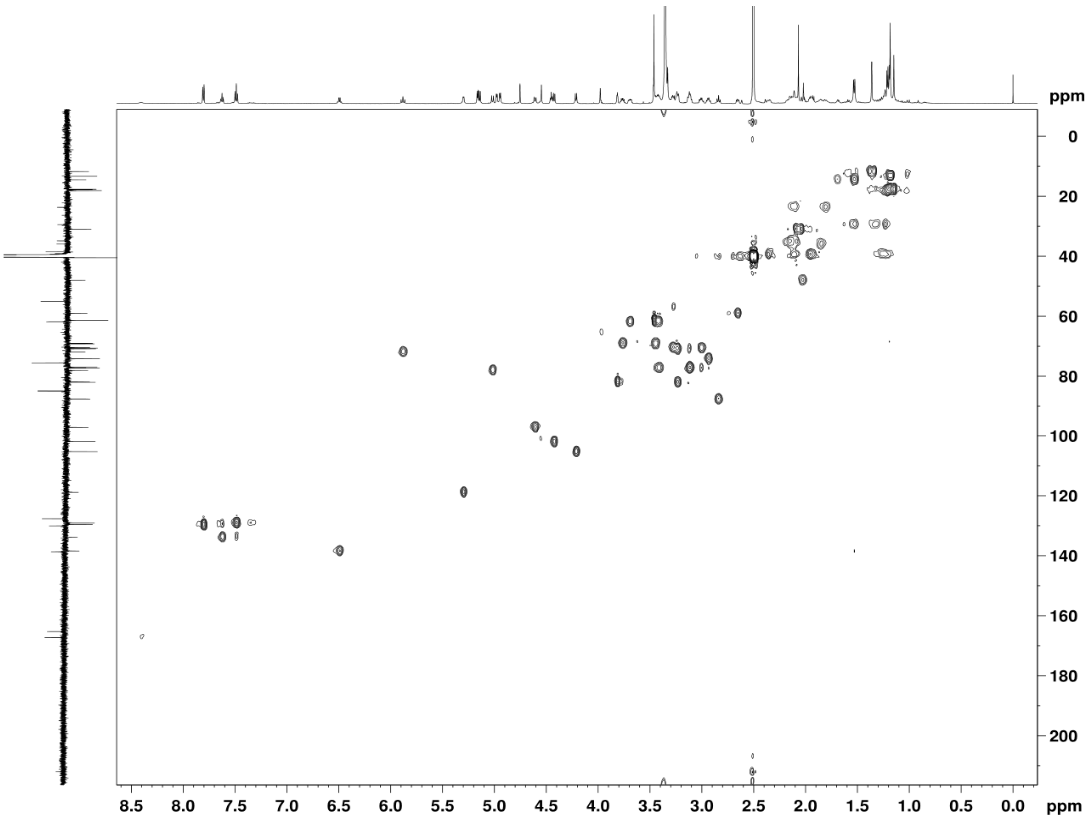

Figure S4. HSQC spectrum of obcordata J (**1**) (DMSO-d_6)

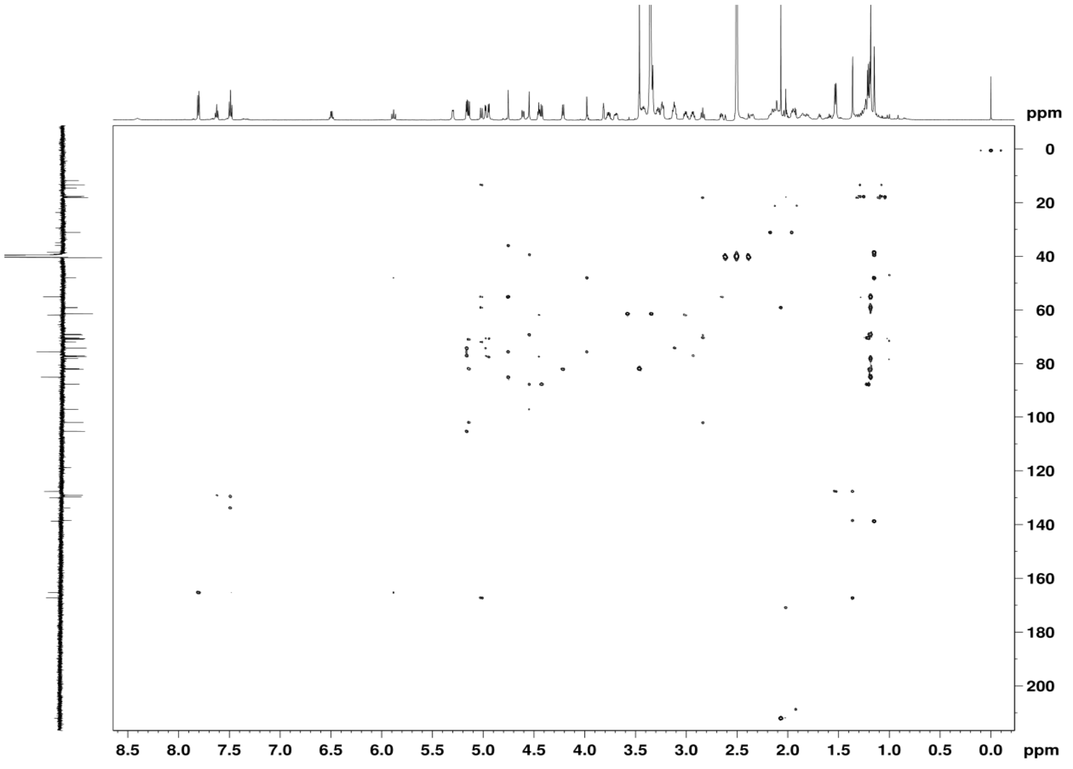

Figure S5. HMBC spectrum of obcordata J (**1**) (DMSO-d_6)

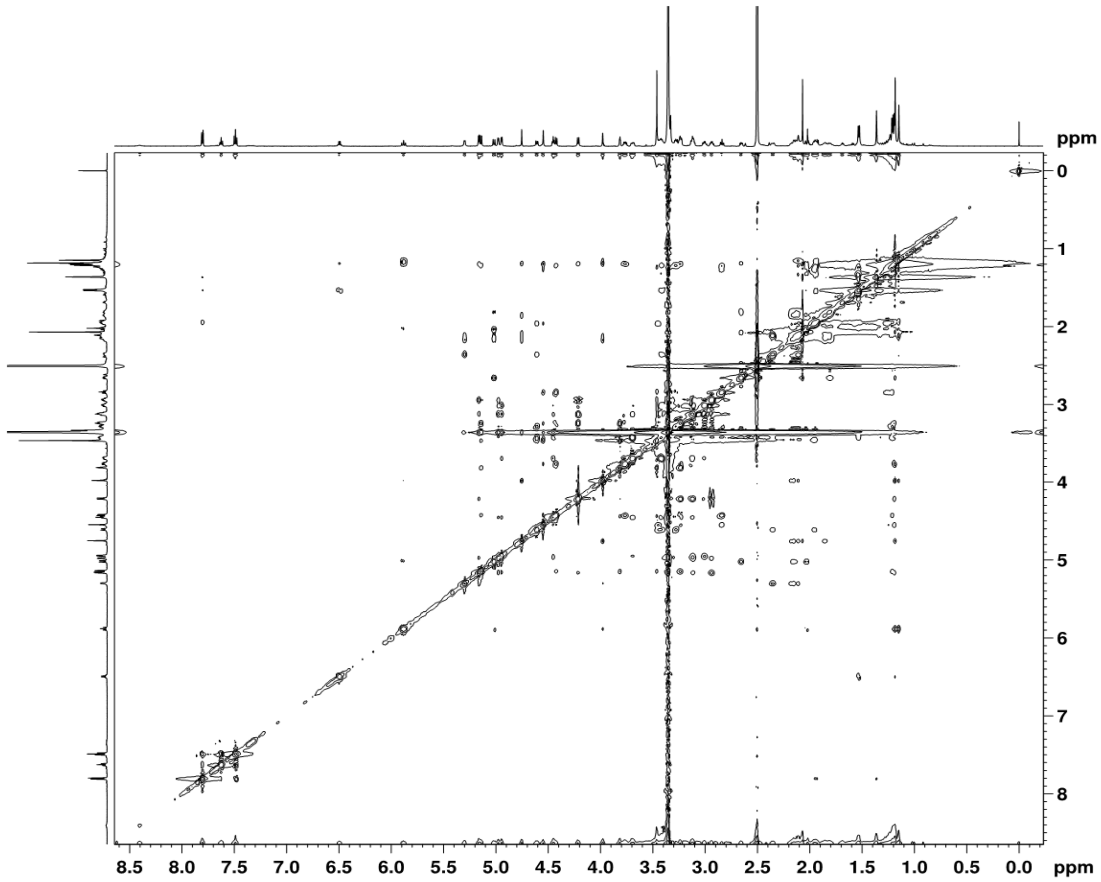

Figure S6. NOESY spectrum of obcordata J (**1**) (DMSO-d_6)

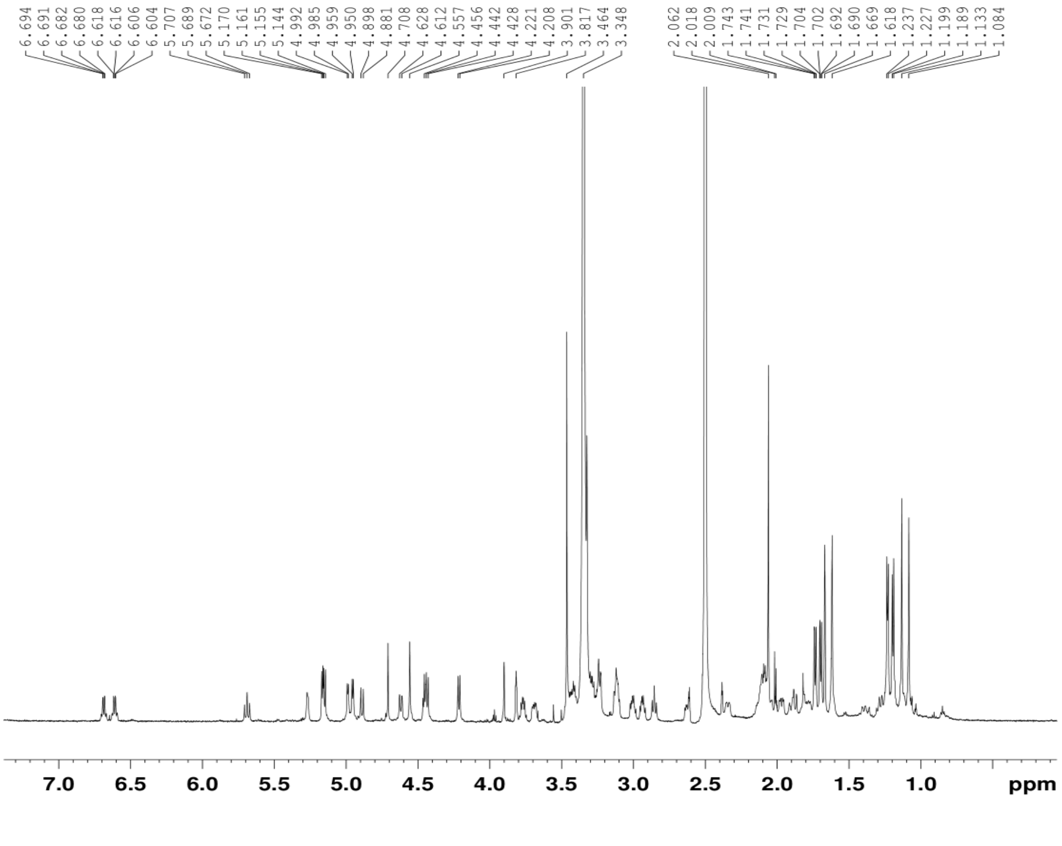

Figure S7. ^{1}H-NMR spectrum of obcordata K (**2**) (600 MHz, DMSO-*d6*)

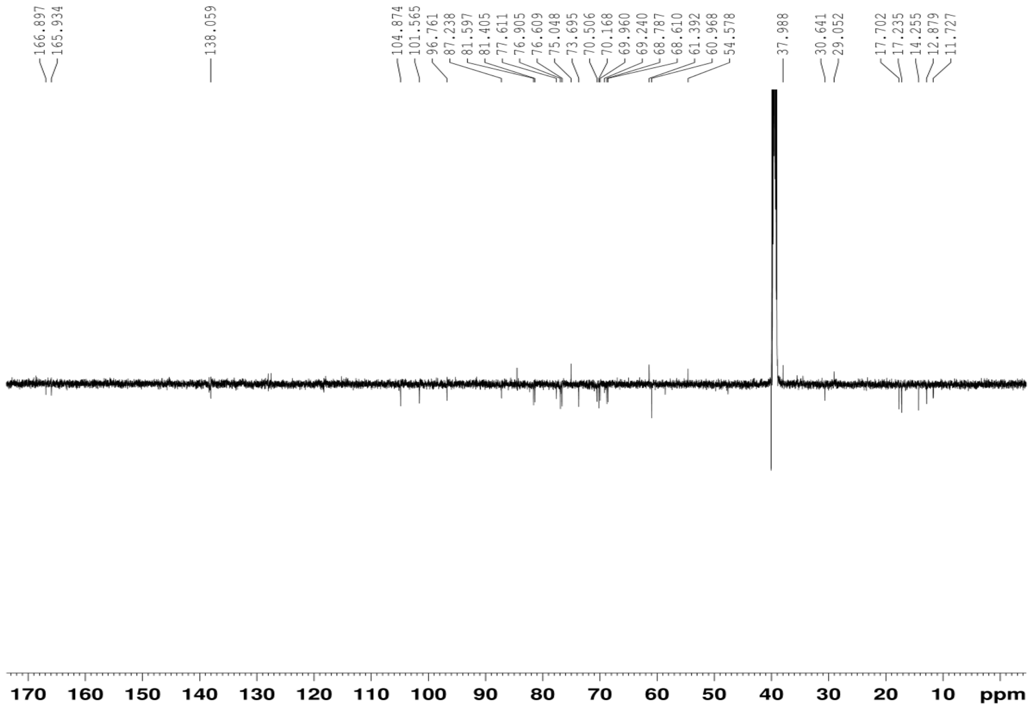

Figure S8. ^{13}C-APT spectrum of obcordata K(**2**) (150 MHz, DMSO-*d6*)

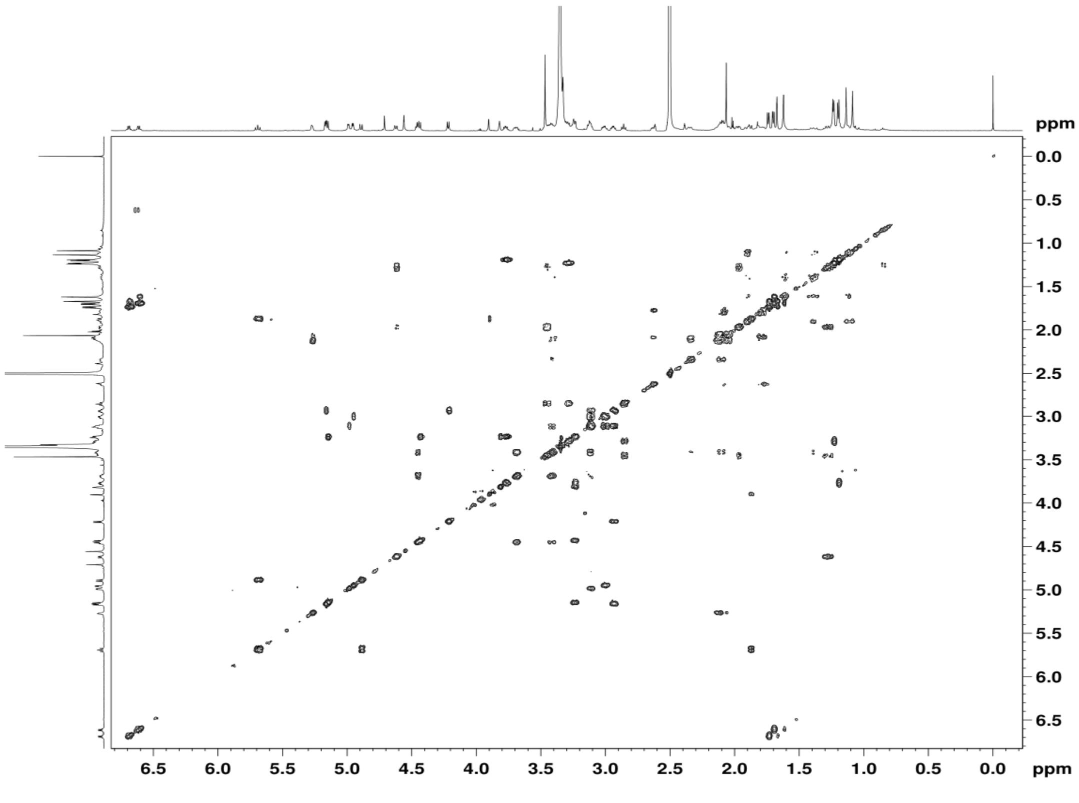

Figure S9. ^{1}H-^{1}H COSY spectrum of obcordata K (**2**) (DMSO-*d6*)

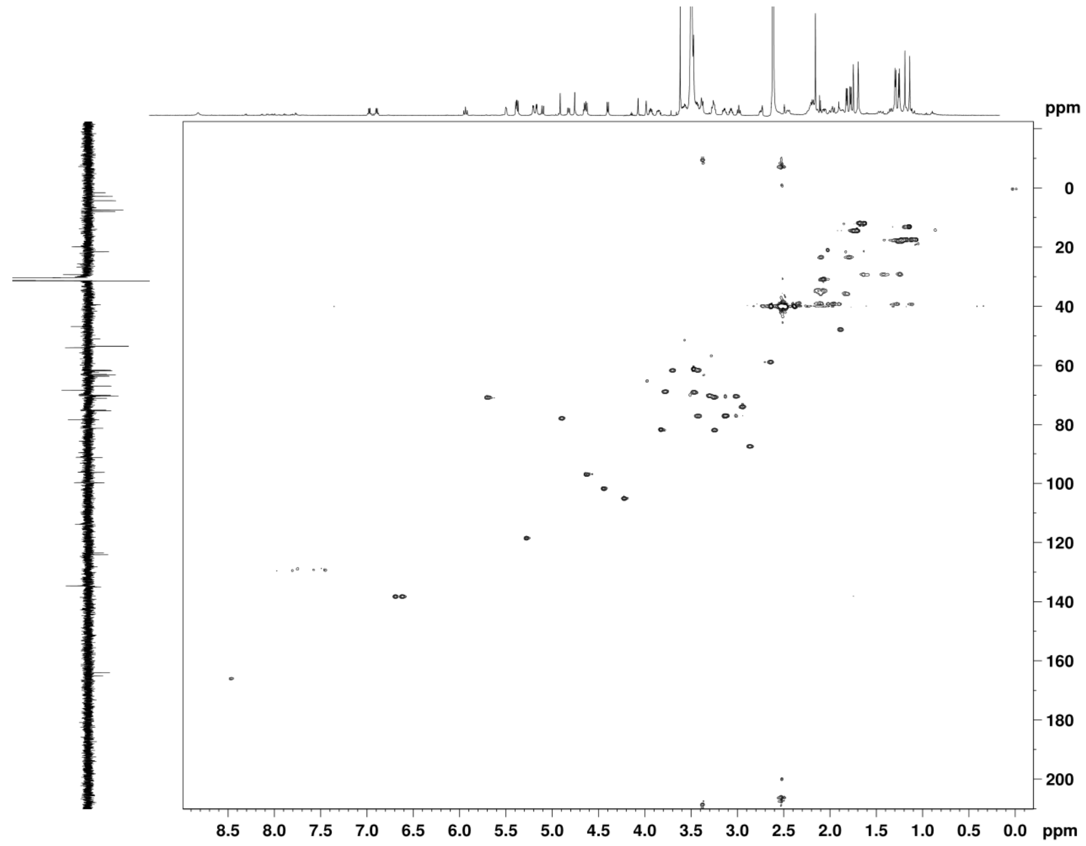

Figure S10. HSQC spectrum of obcordata K (**2**) (DMSO-d_6)

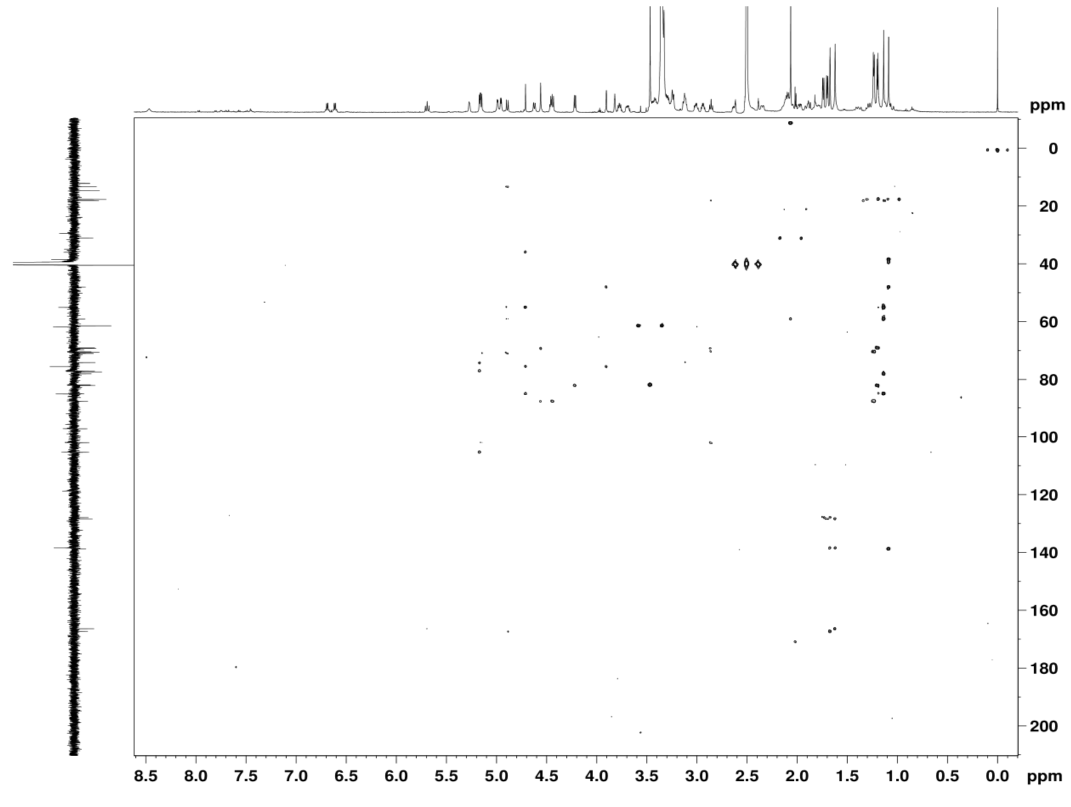

Figure S11. HMBC spectrum of obcordata K (**2**) (DMSO-d_6)

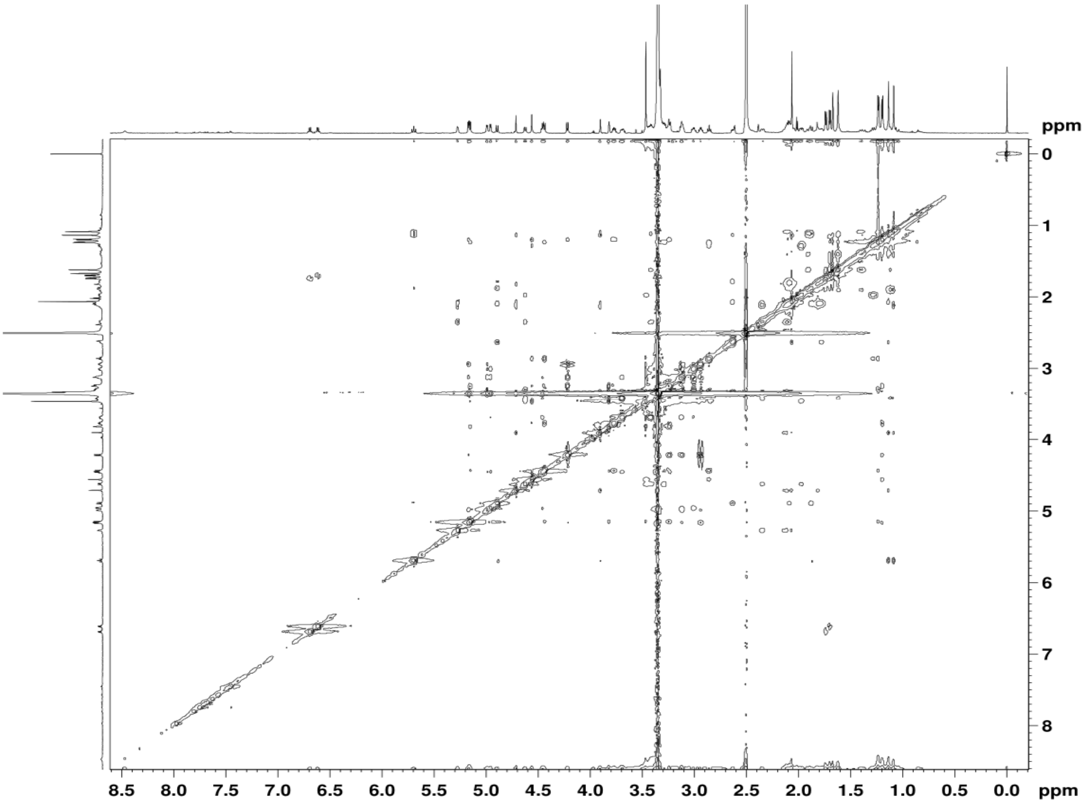

Figure S12. NOESY spectrum of obcordata K (**2**) (DMSO-*d6*)

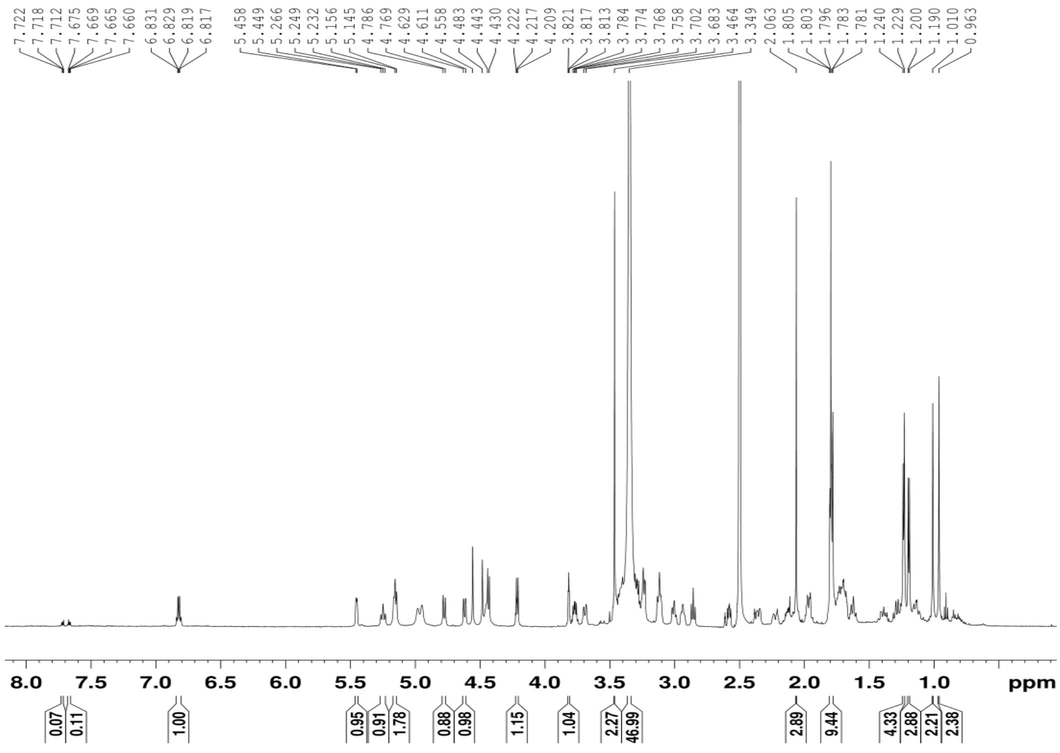

Figure S13. ^{1}H-NMR spectrum of obcordata L (**3**) (600 MHz, DMSO-*d6*)

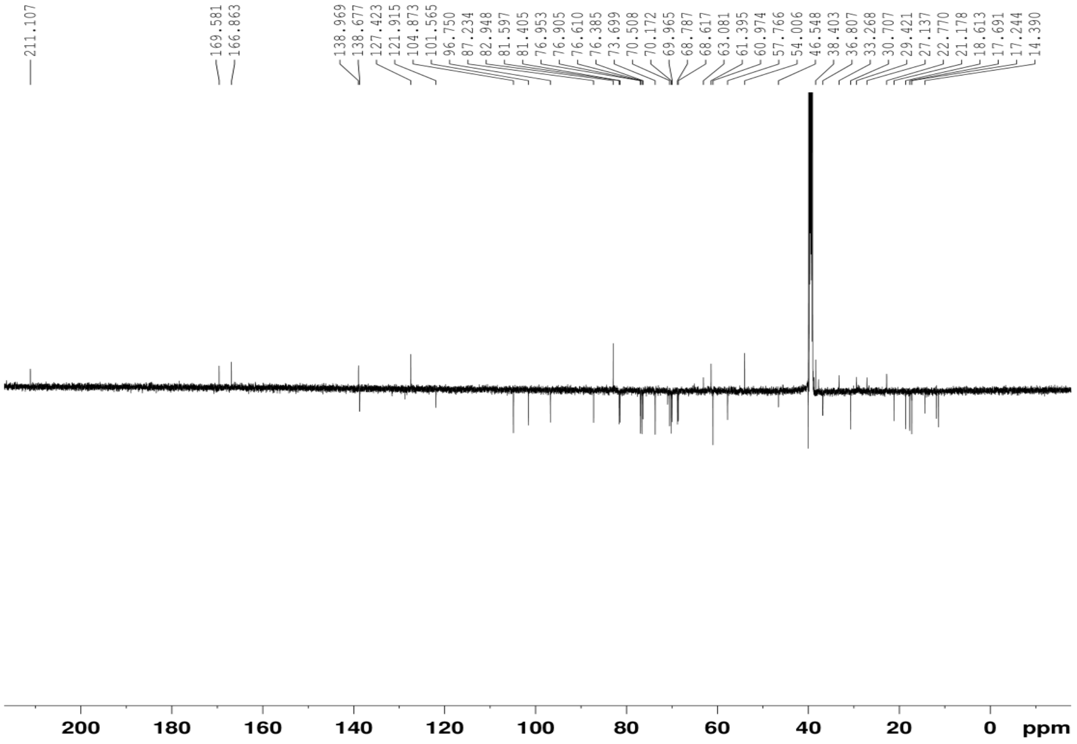

Figure S14. ^{13}C-APT spectrum of obcordata L(**3**) (150 MHz, DMSO-*d6*)

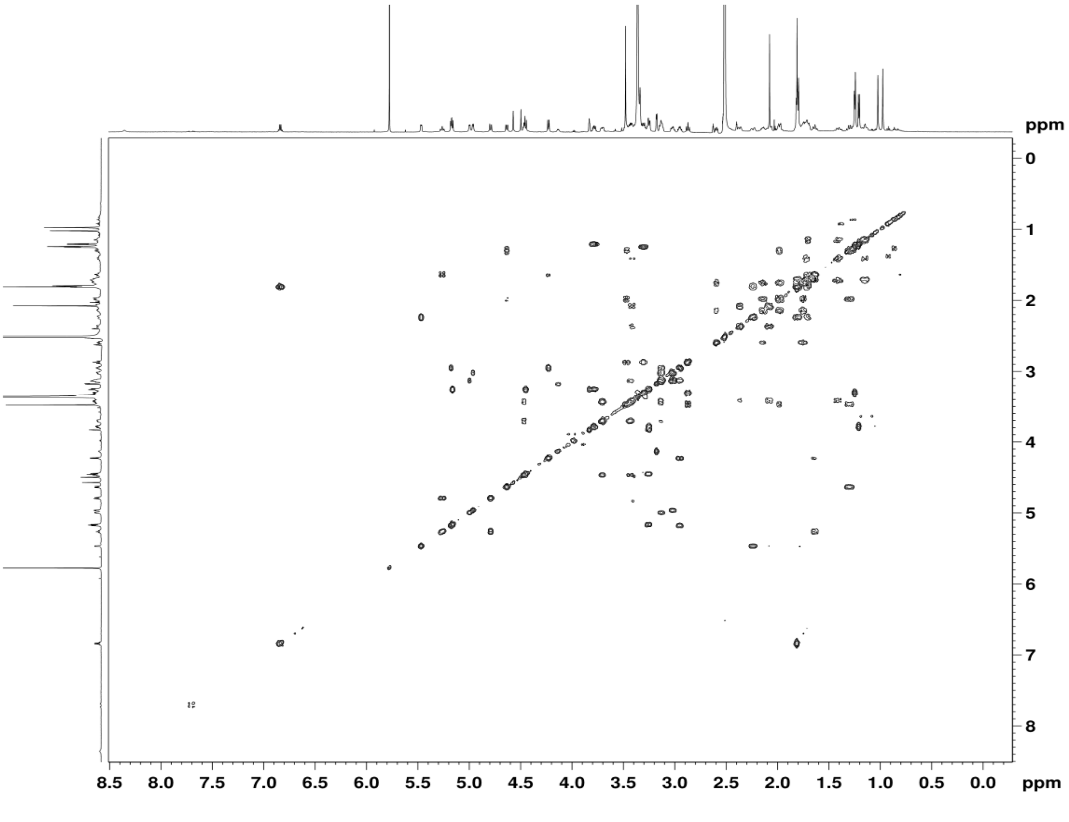

Figure S15. ^{1}H-^{1}H COSY spectrum of obcordata L (**3**) (DMSO-*d6*)

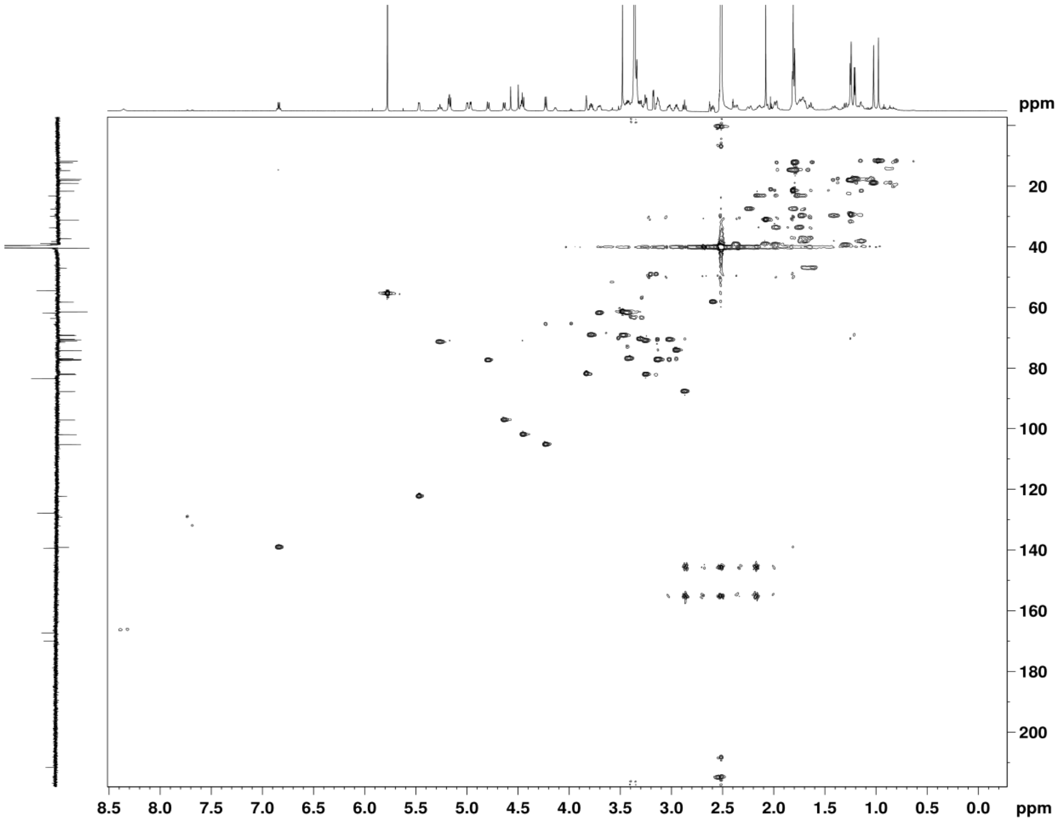

Figure S16. HSQC spectrum of obcordata L (**3**) (DMSO-*d*$_6$)

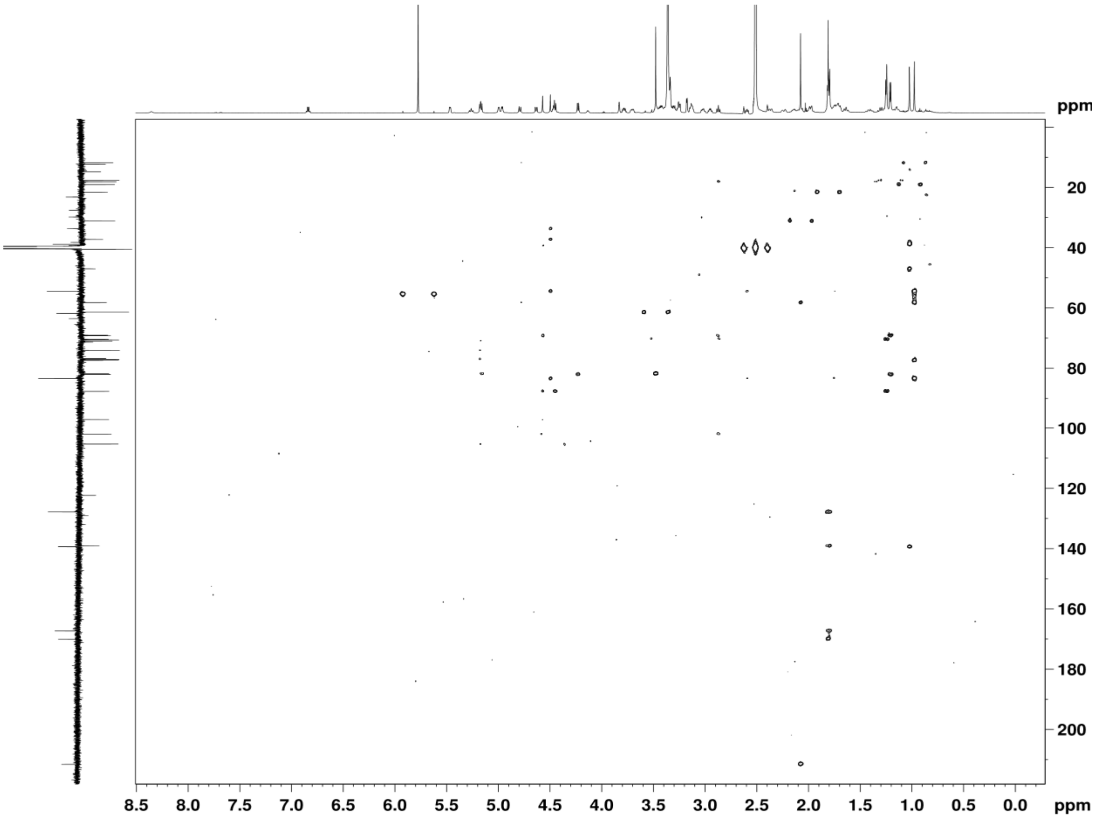

Figure S17. HMBC spectrum of obcordata L (**3**) (DMSO-*d*$_6$)

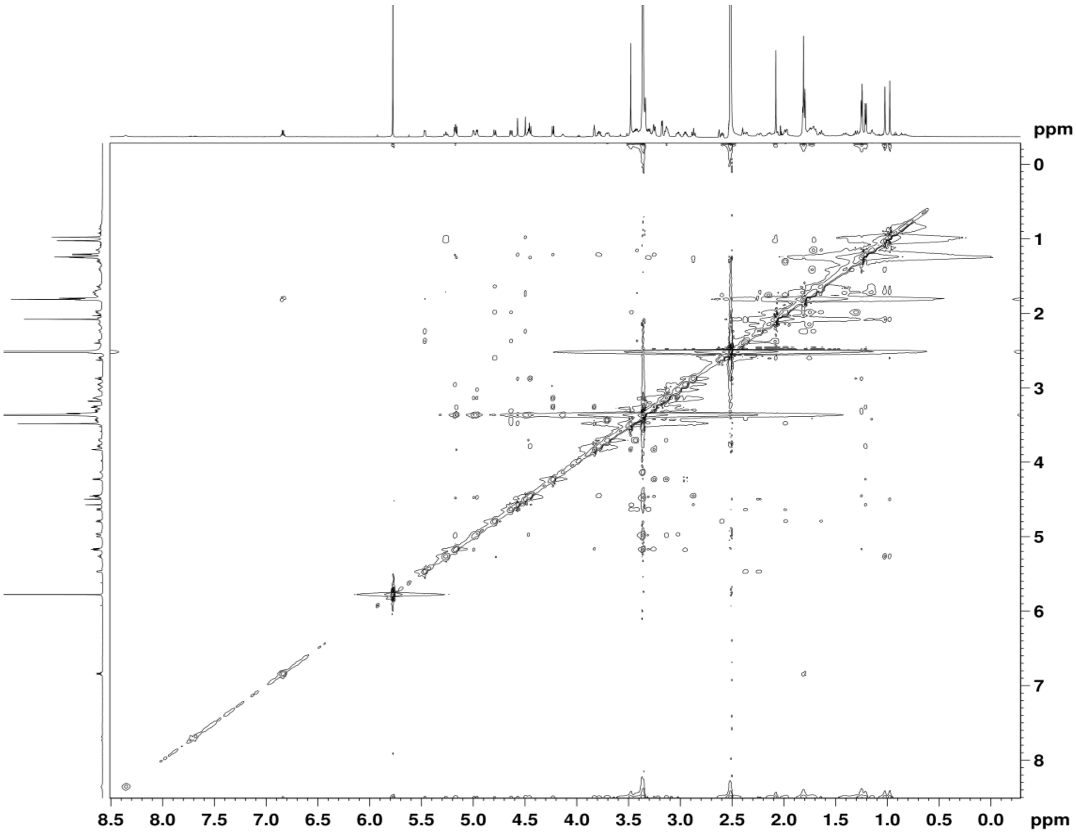

Figure S18. NOESY spectrum of obcordata L (**3**) (DMSO-d_6)

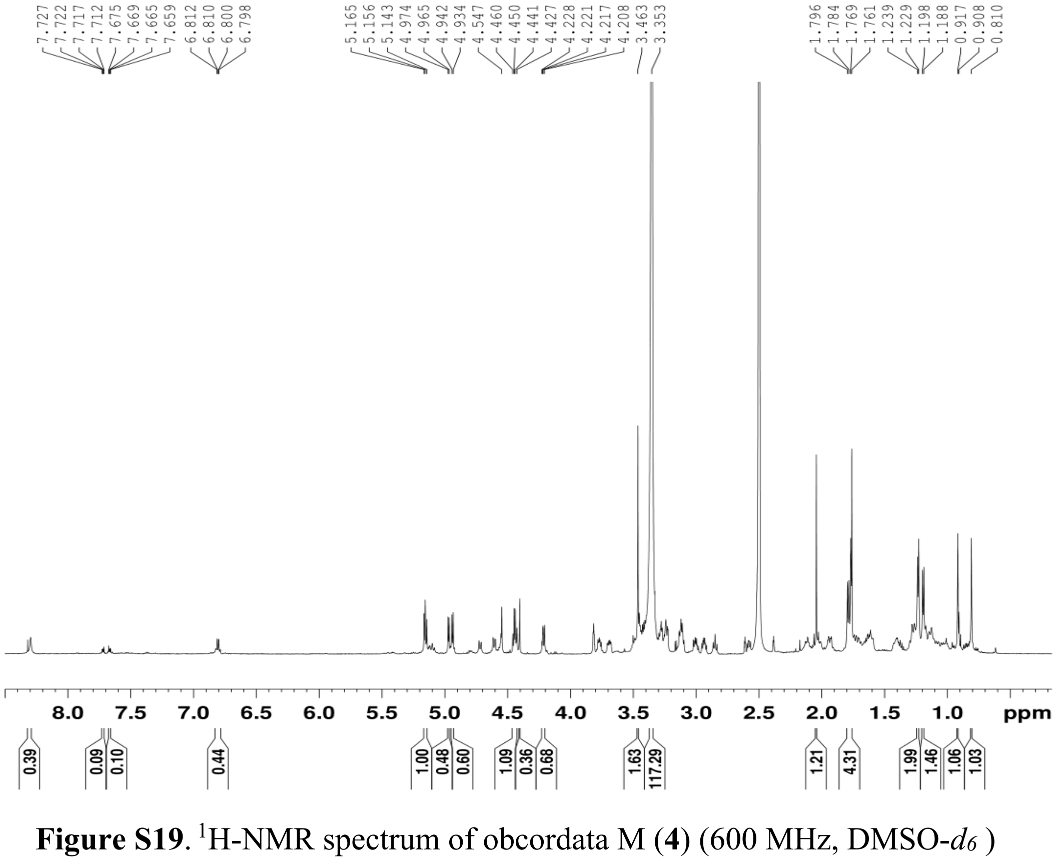

Figure S19. ^{1}H-NMR spectrum of obcordata M (**4**) (600 MHz, DMSO-d_6)

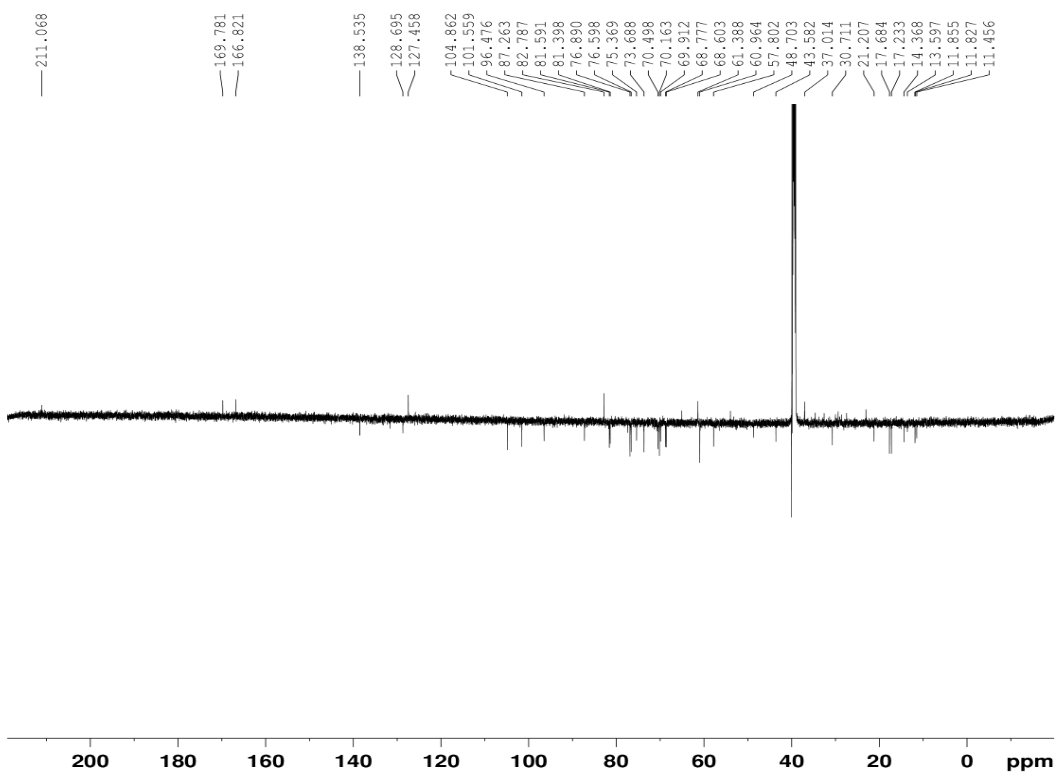

Figure S20. ^{13}C-APT spectrum of obcordata M(**4**) (150 MHz, DMSO-*d6*)

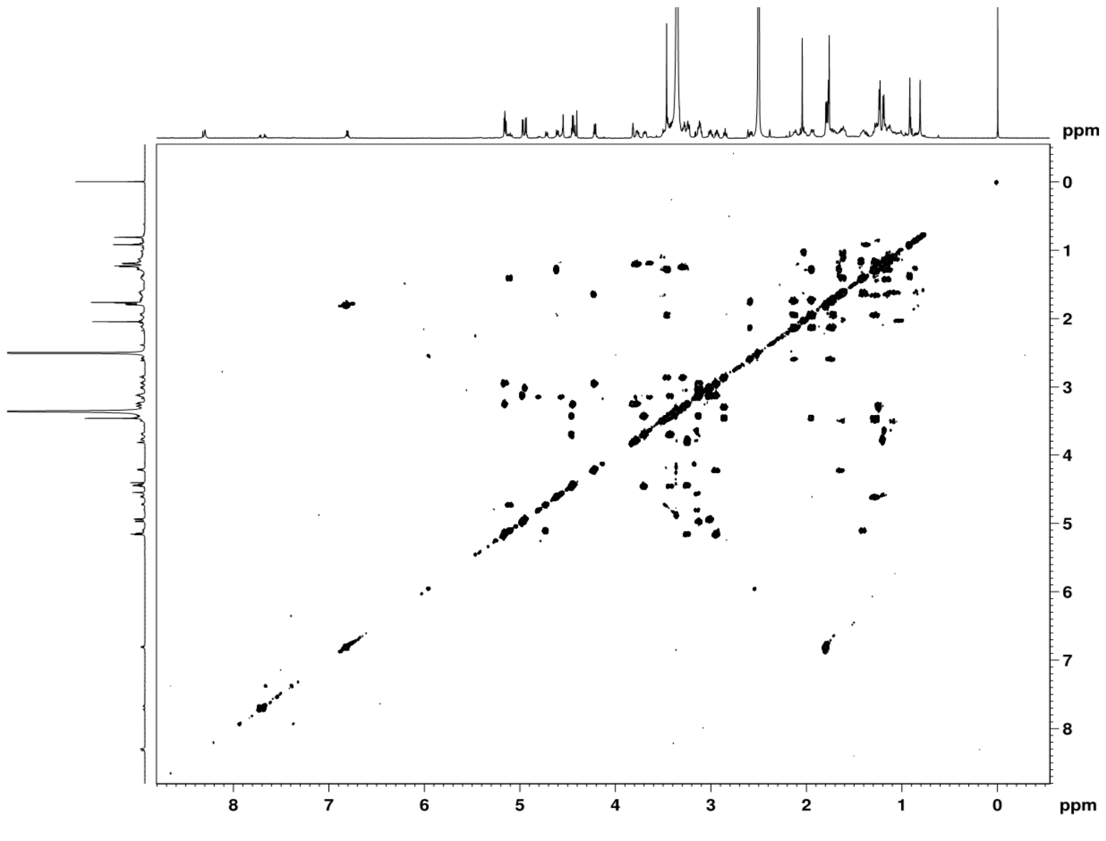

Figure S21. ^{1}H-^{1}H COSY spectrum of obcordata M (**4**) (DMSO-*d6*)

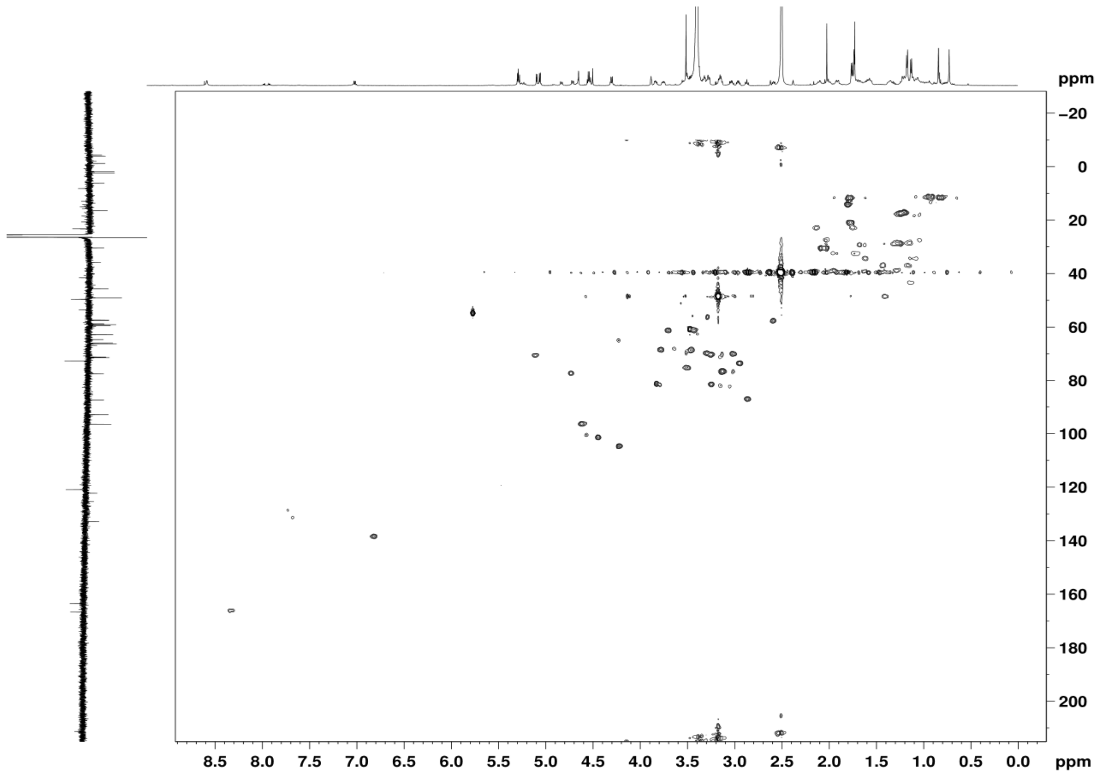

Figure S22. HSQC spectrum of obcordata M (**4**) (DMSO-d_6)

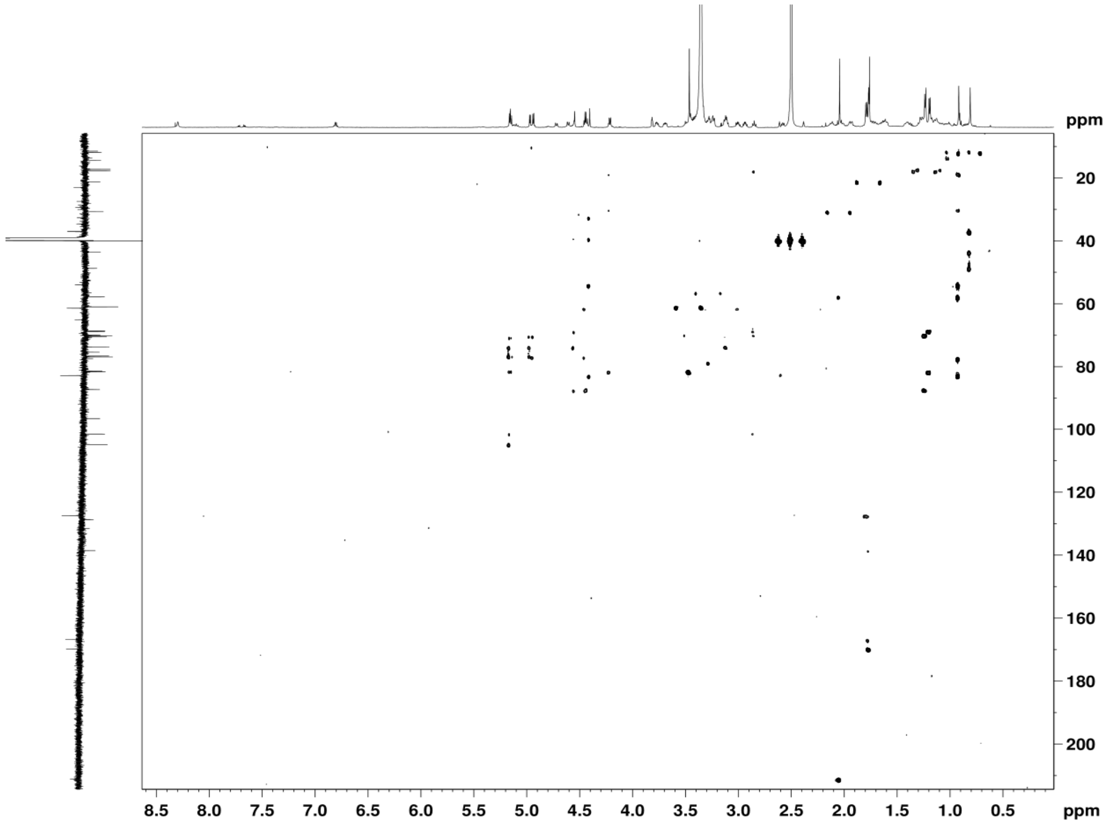

Figure S23. HMBC spectrum of obcordata M (**4**) (DMSO-d_6)

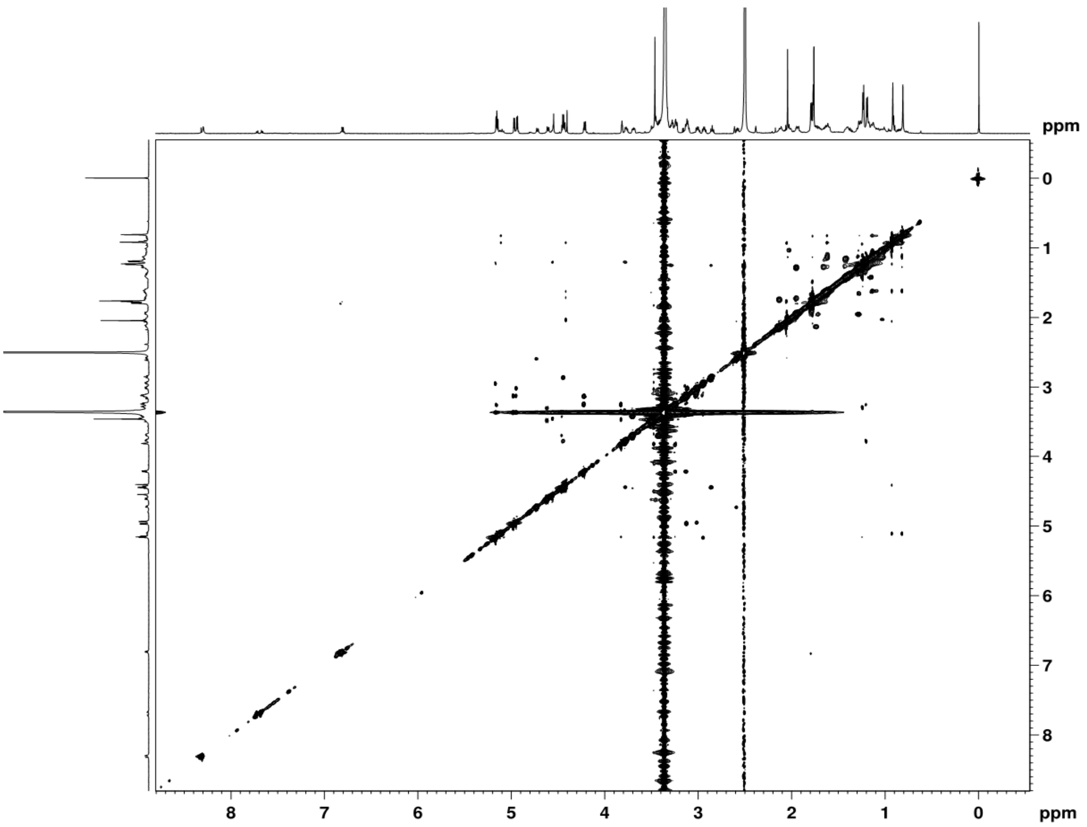

Figure S24. NOESY spectrum of obcordata M (**4**) (DMSO-*d6*)

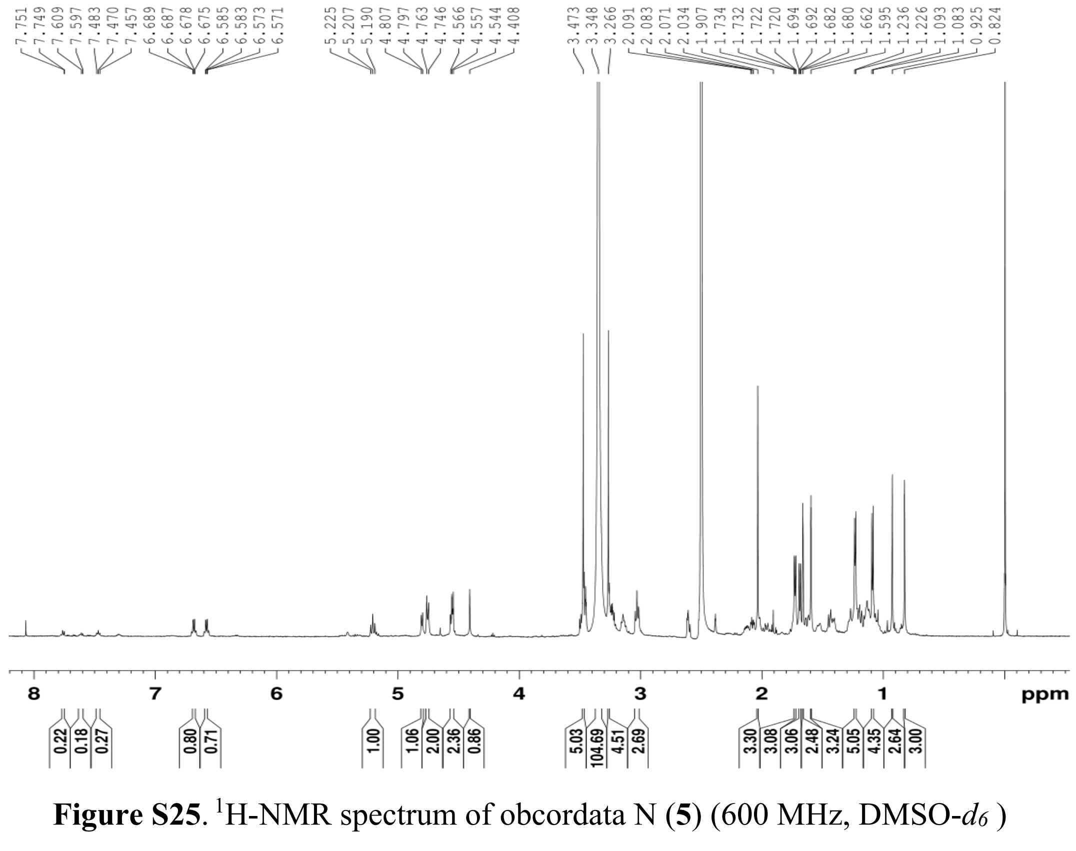

Figure S25. ^{1}H-NMR spectrum of obcordata N (**5**) (600 MHz, DMSO-*d6*)

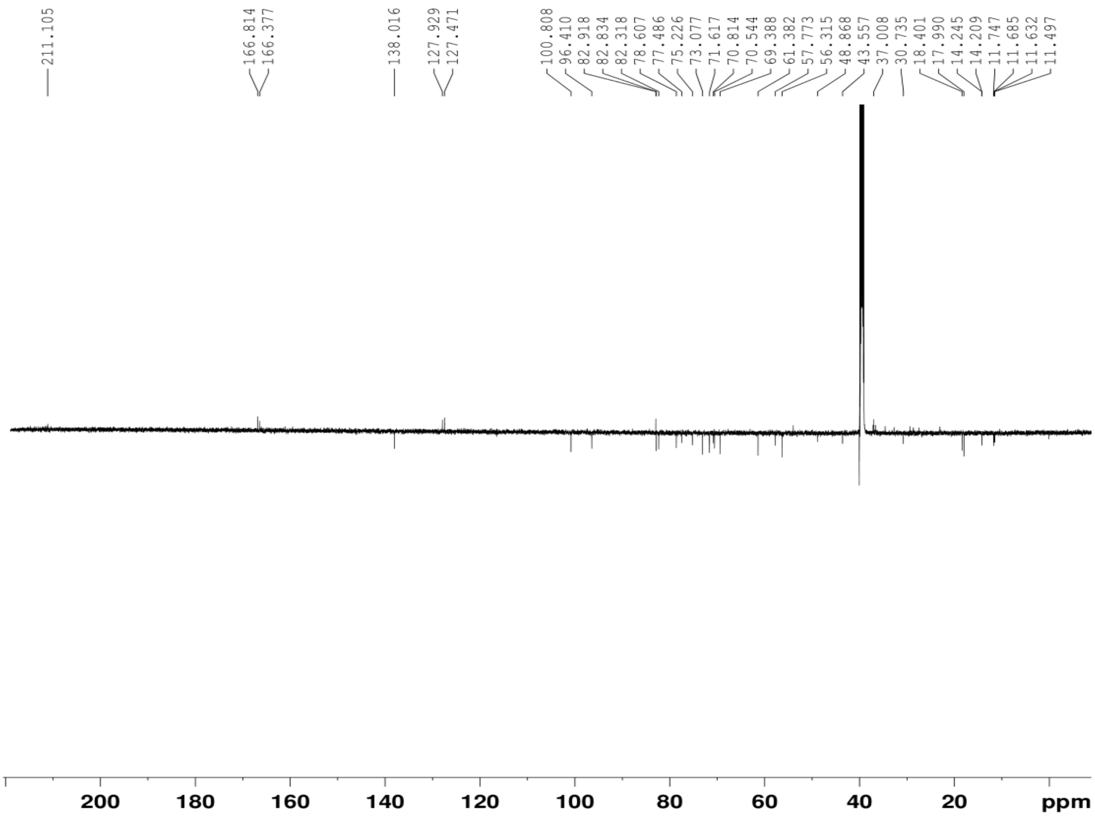

Figure S26. ^{13}C-APT spectrum of obcordata N(**5**) (150 MHz, DMSO-d_6)

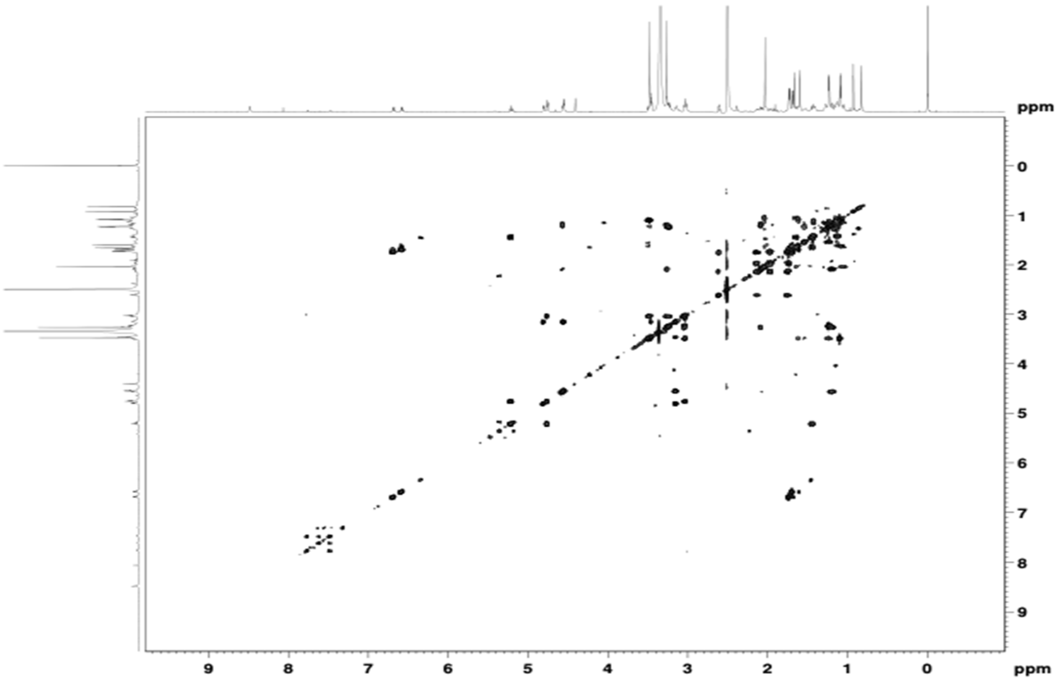

Figure S27. ^{1}H-^{1}H COSY spectrum of obcordata N (**5**) (DMSO-d_6)

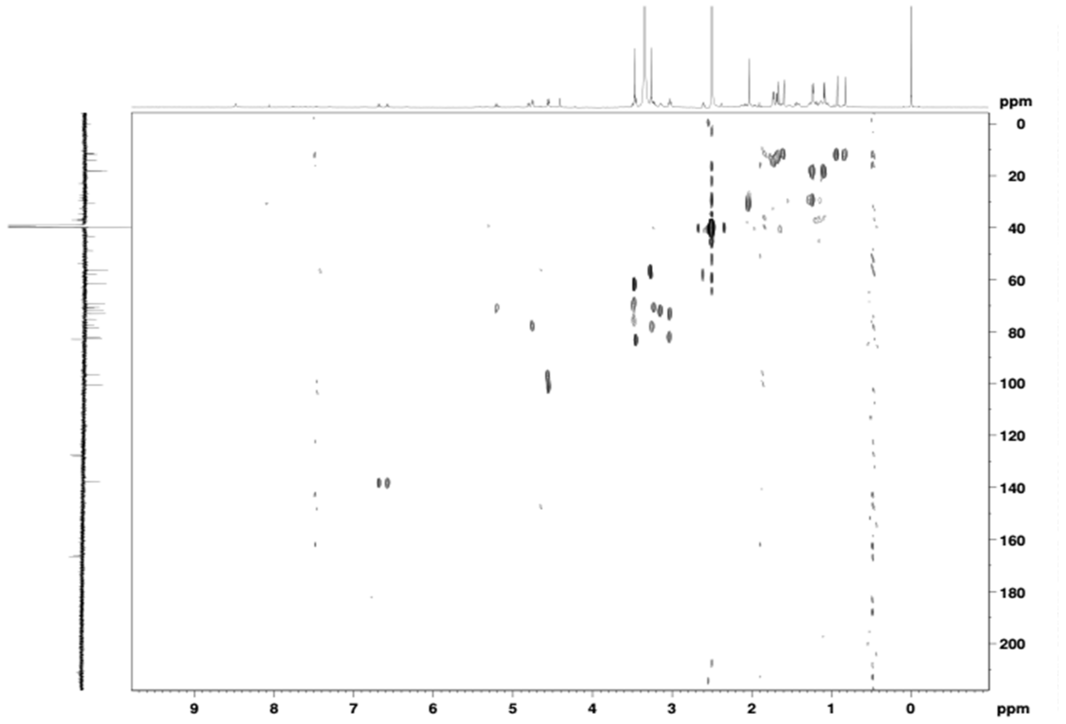

Figure S28. HSQC spectrum of obcordata N (**5**) (DMSO-d_6)

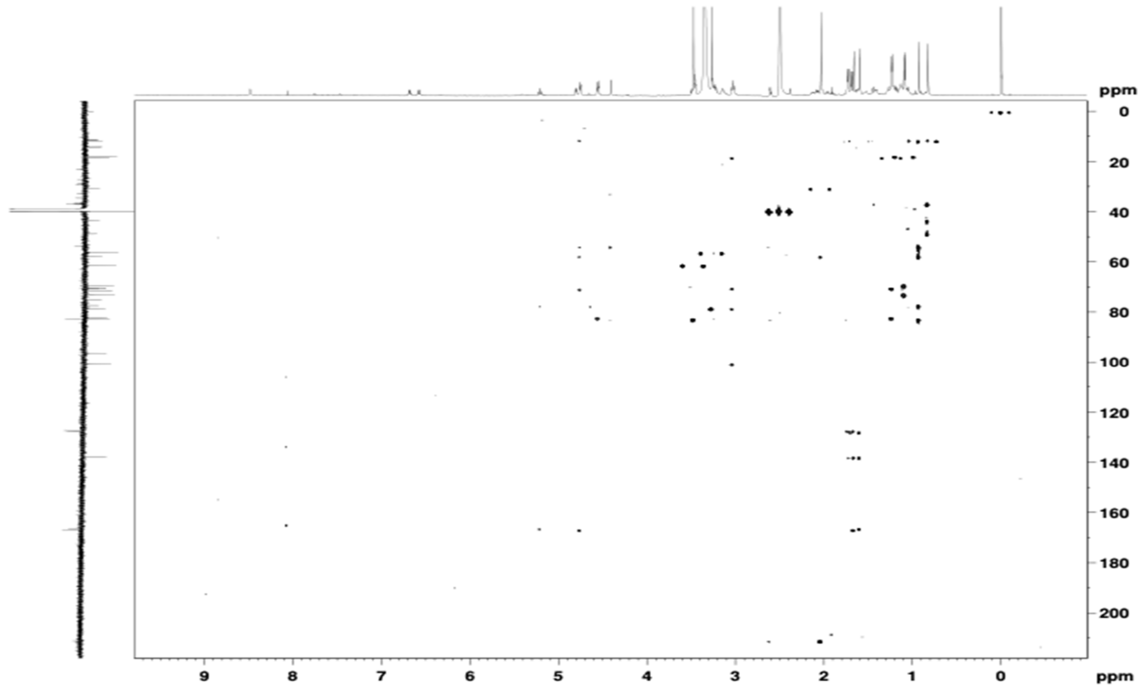

Figure S29. HMBC spectrum of obcordata N (**5**) (DMSO-d_6)

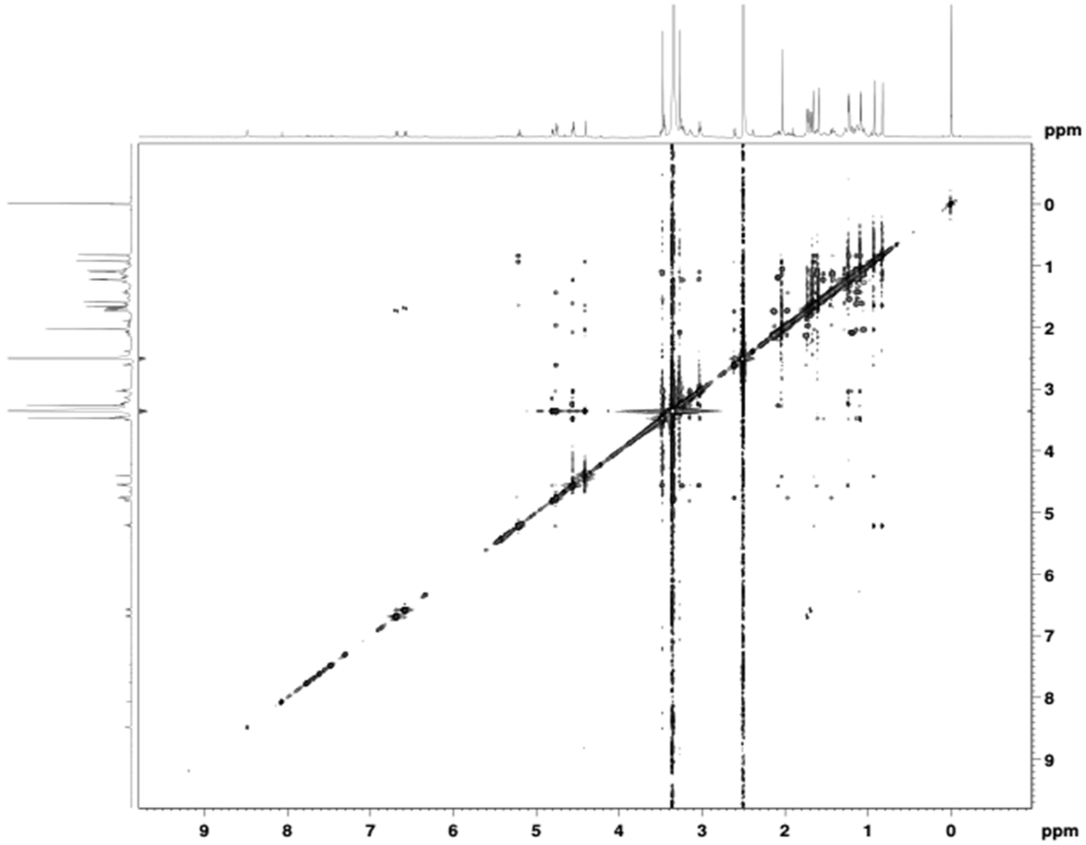

Figure S30. NOESY spectrum of obcordata N (**5**) (DMSO-d_6)
